# Regulation of cyclic electron flow by chloroplast NADPH‐dependent thioredoxin system

**DOI:** 10.1002/pld3.93

**Published:** 2018-11-07

**Authors:** Lauri Nikkanen, Jouni Toivola, Andrea Trotta, Manuel Guinea Diaz, Mikko Tikkanen, Eva‐Mari Aro, Eevi Rintamäki

**Affiliations:** ^1^ Molecular Plant Biology Department of Biochemistry University of Turku Turku Finland

**Keywords:** chloroplast, cyclic electron transfer, fluctuating light, NDH, NTRC, photosynthesis, thioredoxin

## Abstract

Linear electron transport in the thylakoid membrane drives photosynthetic NADPH and ATP production, while cyclic electron flow (CEF) around photosystem I only promotes the translocation of protons from stroma to thylakoid lumen. The chloroplast NADH dehydrogenase‐like complex (NDH) participates in one CEF route transferring electrons from ferredoxin back to the plastoquinone pool with concomitant proton pumping to the lumen. CEF has been proposed to balance the ratio of ATP/NADPH production and to control the redox poise particularly in fluctuating light conditions, but the mechanisms regulating the NDH complex remain unknown. We have investigated potential regulation of the CEF pathways by the chloroplast NADPH‐thioredoxin reductase (NTRC) in vivo by using an Arabidopsis knockout line of *NTRC* as well as lines overexpressing NTRC. Here, we present biochemical and biophysical evidence showing that NTRC stimulates the activity of NDH‐dependent CEF and is involved in the regulation of generation of proton motive force, thylakoid conductivity to protons, and redox balance between the thylakoid electron transfer chain and the stroma during changes in light conditions. Furthermore, protein–protein interaction assays suggest a putative thioredoxin‐target site in close proximity to the ferredoxin‐binding domain of NDH, thus providing a plausible mechanism for redox regulation of the NDH ferredoxin:plastoquinone oxidoreductase activity.

## 1 INTRODUCTION

In their natural habitats, plants face constant fluctuation of light intensity, including both seasonal changes in photoperiod and daily fluctuations according to environmental conditions. Optimization of photosynthesis in plant leaves requires strict balancing between conversion of light energy to chemical energy in photosynthetic light reactions and the energy‐consuming reactions of chloroplast metabolism. Multiple regulatory and photoprotective mechanisms have evolved in photosynthetic organisms to cope with fluctuating light conditions and to prevent the photodamage of both Photosystem (PS) II and PSI (Tikkanen & Aro, 2014; Tikkanen et al., 2012; Tiwari et al., 2016; Townsend, Ware, & Ruban, 2017). Regularly occurring light variations induce long‐term acclimatory changes in the photosynthetic machinery via signaling mechanisms, while temporary fluctuation of light within a day transiently activates short‐term regulatory mechanisms (Armbruster et al., 2014; Bailey, Walters, Jansson, & Horton, 2001; Grieco, Tikkanen, Paakkarinen, Kangasjärvi, & Aro, 2012; Kono & Terashima, 2014). The short‐term mechanisms include nonphotochemical quenching (NPQ), photosynthetic control of electron flow between PSII and PSI, state transitions (ST), cyclic electron flow (CEF), and activation of photosynthetic enzymes both in light and carbon fixation (CBC) reactions (Balsera, Uberegui, Schürmann, & Buchanan, 2014; Demmig‐Adams, Cohu, Muller, & Adams, 2012; Gollan, Lima‐Melo, Tiwari, Tikkanen, & Aro, 2017; Tikkanen & Aro, 2014; Yamori, Makino, & Shikanai, 2016).

Light drives the electron flow from water through PSII, plastoquinone (PQ), cytochrome *b6f*, plastocyanin (PC), and PSI to ferredoxin and ultimately to NADP^+^_,_ producing NADPH. These photosynthetic electron transfer reactions are coupled with ATP synthesis via translocation of protons to the thylakoid lumen, generating a proton gradient over the thylakoid membrane (ΔpH), which together with membrane potential (ΔΨ) constitutes the proton motive force (*pmf*) (Armbruster, Correa Galvis, Kunz, & Strand, 2017; Hangarter & Good, 1982). ΔpH also contributes to induction of the energy‐dependent qE component of NPQ, a photoprotective mechanism that dissipates excess excitation energy from the electron transfer chain (Niyogi & Truong, 2013; Ruban, 2016), and maintains photosynthetic control at Cyt *b6f* (Johnson, 2011; Joliot & Johnson, 2011). Other regulatory mechanisms include the reversible rearrangements of light‐harvesting complexes to balance the excitation of PSII and PSI known as state transitions (Rochaix, 2011; Ruban & Johnson, 2009; Tikkanen et al., 2006) as well as cyclic electron flow around PSI (CEF), a process where electrons are transferred from ferredoxin back to the PQ pool. CEF contributes to the generation of *pmf* and therefore to the production of ATP, and has been suggested to adjust the ATP/NADPH ratio in chloroplasts according to the needs of the CBC (for a recent review, see Yamori & Shikanai, 2016). CEF also provides an alternative electron acceptor mechanism for PSI to relieve stromal overreduction, which is needed to protect the photosystems from damage during early developmental stages of chloroplasts (Allorent et al., 2015; Suorsa, 2015), and during excess illumination or fluctuating light conditions (Miyake, Shinzaki, Miyata, & Tomizawa, 2004; Suorsa et al., 2012; Yamori & Shikanai, 2016; Yamori et al., 2016). CEF has also been shown to be important for controlling the magnitude of the *pmf* (Shikanai & Yamamoto, 2017; Wang, Yamamoto, & Shikanai, 2015), and during induction of photosynthesis (Fan et al., 2007; Joliot & Joliot, 2002). Fan et al. (2007) calculated that CEF contributes a maximum of 68% of total electron flux after a 30‐s illumination of spinach leaves with red and far red light.

Two distinct pathways of CEF have been suggested to exist in plant chloroplasts (Munekage et al., 2004). One CEF pathway involves the chloroplast NADH dehydrogenase‐like complex (NDH), an ortholog of mitochondrial respiratory complex I (Peltier, Aro, & Shikanai, 2016; Shikanai, 2016). However, unlike complex I, which is reduced by NADH, the chloroplast NDH complex is reduced by ferredoxin (Yamamoto, Peng, Fukao, & Shikanai, 2011; Yamamoto & Shikanai, 2013). It has been suggested recently in several studies that CEF via the NDH complex is essential for photosynthesis in low light conditions (Kou, Takahashi, Fan, Badger, & Chow, 2015; Martin, Noarbe, Serrot, & Sabater, 2015; Yamori, Shikanai, & Makino, 2015) as well as for the tolerance of drought (Horvath et al., 2000) and low temperature (Yamori, Sakata, Suzuki, Shikanai, & Makino, 2011). The antimycin A‐sensitive CEF pathway depends on the proteins PROTON GRADIENT REGULATION 5 (PGR5) (Munekage et al., 2002) and PGR5‐LIKE 1 (PGRL1) (DalCorso et al., 2008), and has been suggested to constitute the hypothetical ferredoxin‐plastoquinone reductase (FQR) (Hertle et al., 2013). However, controversy still exists over the molecular identity of FQR and the physiological function of PGR5 (Kanazawa et al., 2017; Leister & Shikanai, 2013; Tikkanen & Aro, 2014). The PGR‐ and NDH‐dependent pathways differ in their energetic properties; two protons per electron are translocated to the lumen (by the Q‐cycle) in the FQR‐pathway, whereas the NDH complex functions as a proton pump and additionally transfers two protons per electron to the lumen (Strand, Fisher, & Kramer, 2017; Strand, Livingston, et al., 2017). A third CEF pathway involving transfer of electrons from ferredoxin or FNR to PQ via heme c_n_ in the Cyt *b*_*6*_ *f* complex has also been proposed (Hasan, Yamashita, Baniulis, & Cramer, 2013). In general, CEF activity is highly dependent on stromal redox state (Breyton, Nandha, Johnson, Joliot, & Finazzi, 2006), and both the PGR‐dependent pathway (Hertle et al., 2013; Strand, Fisher, Davis, & Kramer, 2016) and the NDH pathway (Courteille et al., 2013) have been proposed to be subject to thiol regulation by chloroplast thioredoxins. The physiological roles of each CEF pathway and TRXs involved in the regulation are nevertheless still unclear.

In chloroplasts of Arabidopsis, two thioredoxin systems function in parallel. The ferredoxin–thioredoxin system depends on photosynthetically reduced ferredoxin to supply electrons to the ferredoxin–thioredoxin reductase (FTR), which in turn reduces several thioredoxins, namely TRX‐*f*1 and *f*2, four isoforms of TRX‐*m*, TRX‐*x* as well as TRX‐*y*1 and *y*2 (Schürmann & Buchanan, 2008; Yoshida & Hisabori, 2017). The other system consists of a single enzyme, NADPH‐thioredoxin reductase (NTRC) that contains both a reductase and a thioredoxin domain (Serrato, Perez‐Ruiz, Spinola, & Cejudo, 2004). NTRC is reduced by NADPH, which in addition to the light reactions, is also produced in the oxidative pentose phosphate pathway (OPPP) in darkness. Both chloroplast TRX systems are essential for normal development and growth of plants (Serrato et al., 2004; Wang et al., 2014). The *ntrc* knockout has a stunted and low chlorophyll phenotype, which is particularly severe in plants grown under short photoperiods (Lepistö et al., 2009, 2013; Pérez‐Ruiz et al., 2006). The mutant suffers from an impaired ability to activate the ATP synthase and CBC enzymes as well as elevated nonphotochemical quenching (NPQ) (Carrillo, Froehlich, Cruz, Savage, & Kramer, 2016; Naranjo et al., 2016; Nikkanen, Toivola, & Rintamäki, 2016; Thormählen et al., 2017). In contrast, NTRC overexpression lines (OE‐NTRC), with 15–20 times higher NTRC content compared to WT, show wild‐type–like visible phenotype, enhanced vegetative growth, and increased activation of the ATP synthase and CBC enzymes, particularly in darkness and low light (Nikkanen et al., 2016; Toivola et al., 2013). NTRC has a less negative midpoint redox potential than FTR (Hirasawa et al., 1999; Yoshida & Hisabori, 2016) and plays an important regulatory role under low irradiance, while the FTR‐dependent system probably requires more extensive illumination to be fully activated (Geigenberger, Thormählen, Daloso, & Fernie, 2017; Nikkanen et al., 2016; Thormählen et al., 2017). Recent studies have revealed a significant functional overlap and crosstalk between the two chloroplast TRX systems, and indicated that they cooperatively regulate ATP synthesis, the CBC, starch synthesis, and scavenging of reactive oxygen species (ROS) (Geigenberger et al., 2017; Nikkanen et al., 2016; Pérez‐Ruiz, Naranjo, Ojeda, Guinea, & Cejudo, 2006; Thormählen et al., 2015).

Here, we have used NTRC overexpression lines as well as the *ntrc* knockout mutant of *Arabidopsis thaliana* to investigate the potential role of the NTRC system in regulating CEF. Our results emphasize the important role of thioredoxins in the chloroplast regulatory network, particularly controlling the photosynthetic redox balance under fluctuating light conditions. We suggest that NTRC plays a crucial role in the activation of the NDH‐dependent electron flow in darkness (chlororespiration) and during dark to light transitions. Overexpression of NTRC, on the other hand, maintains constant NDH‐CEF activity leading to elevated *pmf* and improved utilization of light energy under fluctuating light conditions. Our results also suggest that NTRC does not activate the PGR‐dependent CEF, but contributes to the PGR5‐dependent downregulation of thylakoid membrane proton conductivity upon transient exposure of leaves to high light intensity. Through control of both CEF and the activity of the ATP synthase, NTRC would play a pivotal role in adjusting the proton motive force and photosynthetic redox poise in Arabidopsis chloroplasts.

## 2 RESULTS

### 2.1 NTRC is an active reductant in darkness and low light conditions

**Figure 1:**
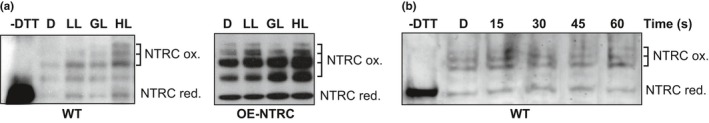
In vivo redox state of NTRC in dark‐adapted and illuminated leaves. (a) Total protein extract was isolated from WT and OE‐NTRC leaves incubated in darkness (D), or illuminated for 2 hr in low light (LL, 40 μmol photons m^−2^ s^−1^), growth light (GL, 200 μmol photons m^−2^ s^−1^) or high light (HL, 800 μmol photons m^−2^ s^−1^). Free thiols of proteins were blocked with NEM, disulfides reduced with DTT and newly formed thiols alkylated with MAL‐PEG. The in vivo‐reduced form of NTRC therefore migrates faster in SDS‐PAGE than the in vivo‐oxidized forms. –DTT stands for the unlabeled control sample where DTT was not added after incubating the leaf extracts in a buffer containing NEM. Protein content of samples has been equalized only based on the amount of starting leaf material, and the apparent differences in band intensity should not be taken as an indication of differences in NTRC content between light treatments. For an analysis of the origin of different MAL‐PEG–labeled bands see Supporting Information Figure S1. (b) NTRC redox state in WT during a transition from dark to growth light. Samples were taken from darkness (2 hr) (D) and 15, 30, 45, and 60 s after onset of illumination

The NADPH produced in the oxidative pentose phosphate pathway (OPPP) has been proposed to maintain the NTRC pool partially reduced, and thus active in darkness and when low irradiance limits photosynthesis (Geigenberger et al., 2017; Pérez‐Ruiz et al., 2006). To test this hypothesis, we analyzed the in vivo redox state of NTRC by a mobility shift assay using the WT or OE‐NTRC protein extracts alkylated with methoxypolyethylene glycol maleimide (MAL‐PEG). The assays indicated that the redox state of the NTRC pool remains fairly constant in all light intensities and during dark‐to‐light transitions, with a significant proportion of the enzyme pool in fully or partially reduced form (Figure 1a,b). This is also the case in OE‐NTRC, despite a 20‐fold increase in the NTRC content of leaves (Figure 1 and Supporting Information Figure S1).

### 2.2 NDH‐dependent CEF is enhanced by overexpression of NTRC

**Figure 2:**
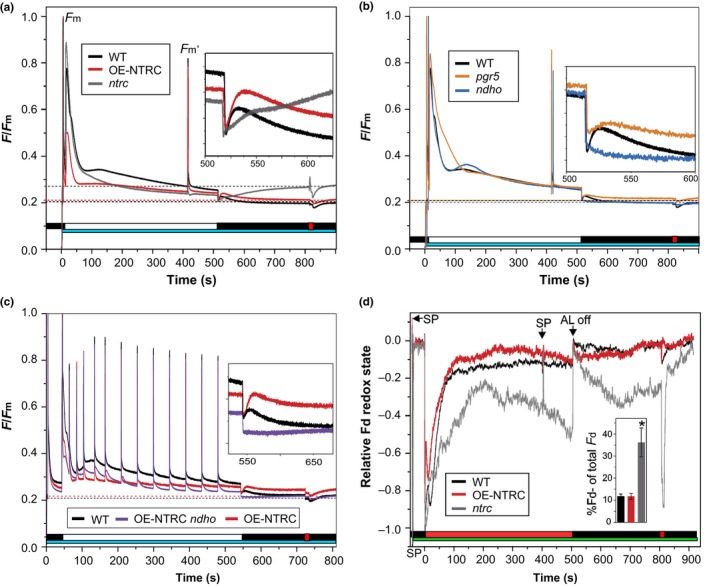
Postillumination fluorescence rise (PIFR) in dark‐adapted leaves. (a) and (b) PIFR was measured from WT, OE‐NTRC,* ntrc* (a), *pgr5* and *ndho* (b) leaves. The smaller windows show magnifications of the ~100 s of the PIFR. The cyan bars indicate exposure to a 480 nm measuring light of 0.28 μmol photons m^−2^ s^−1^, the white bar depicts illumination with 67 μmol photons m^−2^ s^−1^ white light and the red bar shows the duration of a pulse of far red light. The dashed lines indicate the F_0_ values of the lines. The curves are averages of measurements from three to seven individual leaves, and are normalized to Fm. (c) PIFR in WT, OE‐NTRC, and OE‐NTRC *ndho*. PIFR was measured as in (a) and (b), but saturating pulses were administered with 20‐s intervals for the first minute, 30‐s interval for the second minute, and 45‐s intervals for the rest of the experiment. Representative curves of three to four measurements of individual leaves are shown. (d) Fd redox changes during and after illumination of dark‐adapted WT,* ntrc*, and OE‐NTRC leaves. The Fd redox changes were deconvoluted from four near‐infrared absorbance differences measured with a Dual/Klas‐NIR spectrometer according to Klughammer and Schreiber (2016). Leaves were illuminated at actinic red light (630 nm) of 61 μmol photons m^−2^ s^−1^. The red bar shows the duration of a pulse of far red light, the green bar the duration of the four measuring beams, the black bar the duration of the dark period after illumination, SP means saturating pulse. Averages of the relative amount of reduced Fd at the end of actinic illumination ± *SE* in three to seven individual leaves are shown in the column chart inside the figure. Representative curves of WT, OE‐NTRC, and *ntrc* are shown in the figure

In order to determine the effect of altered chloroplast thiol redox state on the activity of NDH‐dependent CEF, we measured the postillumination rise of chlorophyll a fluorescence (PIFR). The PIFR has been suggested to represent electron flow from stromal reductants via the NDH complex to the plastoquinone (PQ) pool upon cessation of illumination (Gotoh, Matsumoto, Ogawa, Kobayashi, & Tsuyama, 2010; Shikanai et al., 1998). The OE‐NTRC line showed a significantly larger PIFR after preillumination with low intensity white light than WT, suggesting increased CEF activity (Figure 2a). No PIFR was detected in the *ndho* mutant, which is lacking a functional NDH complex (Rumeau et al., 2005). A diminished PIFR was also observed in the *pgr5* line, which is deficient in PGR‐dependent CEF (Munekage et al., 2002) (Figure 2b), but this decrease in PIFR is likely due to the lower content of reduced ferredoxin in *pgr5* chloroplasts because of impaired electron transfer from PSI (Takagi & Miyake, 2018; Tiwari et al., 2016). These results suggest that NTRC contributes to the activation of NDH‐dependent CEF. In order to confirm that the increased PIFR in OE‐NTRC derives from the activity of the NDH complex, we generated an NTRC overexpression line in the *ndho* mutant background (OE‐NTRC *ndho*), which indeed was fully missing the PIFR (Figure 2c). Additionally, the relaxation of fluorescence after saturating pulses during actinic illumination was delayed in OE‐NTRC *ndho* (Figure 2c). The level of NTRC overexpression in OE‐NTRC *ndho* plants was confirmed by immunoblotting and found to be in the same range as the OE‐NTRC line (Supporting Information Figure S1B).

The *ntrc* knockout exhibited a slower initial PIFR response after illumination, but the PIFR did not decline after 15–20 s in darkness as in WT or OE‐NTRC. Instead, the PIFR continued to rise throughout the duration of the dark phase of the experiment (Figure 2a). A brief pulse of far red (FR) light quenched the fluorescence, but after cessation of the FR light, fluorescence quickly rose back to its high pre‐FR level. The F_0_ level was elevated in dark‐adapted *ntrc* leaves in comparison to WT, OE‐NTRC, or other mutant lines (Figure 2a). The abnormal fluorescence pattern in Figure 2a may also be due to the highly pleiotropic phenotype of the *ntrc* mutant, particularly when grown under a short‐day photoperiod. The *ntrc* knockout had high NPQ in the experimental conditions (Supporting Information Figure S2B), and its relaxation in darkness likely contributed to the PIFR. Moreover, the *ntrc* mutant has an impaired capacity to scavenge hydrogen peroxide (H_2_O_2_) (Kirchsteiger, Pulido, Gonzalez, & Javier Cejudo, 2009; Pulido et al., 2010), whose accumulation has been shown to cause an increase in NDH‐dependent CEF (Strand et al., 2015; Strand, Livingston, et al., 2017). In order to clarify whether the differences in PIFR were caused indirectly by metabolic disturbances due to impaired growth in a short photoperiod and/or accumulation of H_2_O_2_, we estimated the levels of H_2_O_2_ in illuminated WT, *ntrc*, and OE‐NTRC leaves by DAB staining, and repeated the PIFR‐experiment with plants grown in a 12 hr/12 hr photoperiod. An increased amount of H_2_O_2_ was detected in both low light‐ and high light‐treated *ntrc* leaves in comparison to WT (Supporting Information Figure S2C). No difference was observed in the amount of H_2_O_2_ between OE‐NTRC and WT (Supporting Information Figure S2C), indicating that the elevation of PIFR in OE‐NTRC (Figure 2a) is not caused by increased content of H_2_O_2_. Furthermore, the PIFR mostly disappeared in a 12‐hr photoperiod‐grown *ntrc,* but remained similar to 8‐hr photoperiod‐grown plants in WT and OE‐NTRC (Supporting Information Figure S2A).

Altered PIFR responses in OE‐NTRC and *ntrc* could hypothetically be caused by changes in the available amount of reduced ferredoxin (Fd^−^), the substrate of the NDH complex (Yamamoto & Shikanai, 2013; Yamamoto et al., 2011). In order to investigate this possibility, we used the Dual/Klas‐NIR spectrometer, which allows deconvolution of the Fd signal from plastocyanin (PC) and P700 signals (Klughammer & Schreiber, 2016; Schreiber, 2017; Schreiber & Klughammer, 2016), to measure the redox state of Fd in WT, *ntrc*, and OE‐NTRC under similar light and postillumination conditions as used in the PIFR measurements. In OE‐NTRC, reduced Fd was reoxidized slightly faster than in WT upon onset of illumination, but upon cessation of actinic illumination, there was no difference in the fraction of reduced Fd between WT and OE‐NTRC (~11% in both lines, Figure 2d). In *ntrc* leaves, however, almost 40% of the Fd pool was reduced at the end of actinic illumination, and a significantly increased reduction of Fd also occurred in darkness after initial oxidation (Figure 2d). These results indicate that the elevated PIFR in OE‐NTRC is not caused by increased accumulation of substrate of the NDH complex. In contrast, the PIFR signal in *ntrc* is very likely partially caused by an increase in the relative amount of reduced Fd at the end of illumination.

**Figure 3:**
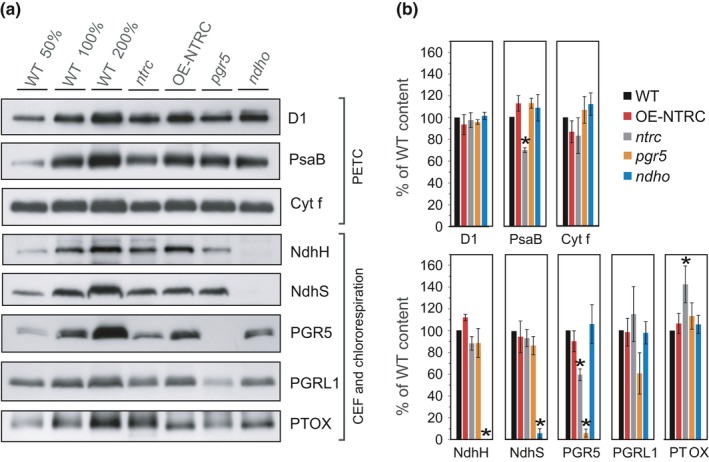
Content of proteins functioning in the photosynthetic electron transfer chain (PETC), cyclic electron flow (CEF), and chlororespiration in WT,* ntrc*, OE‐NTRC,* pgr5,* and *ndho*. (a) Representative immunoblots showing the content of D1, PsaB, Cyt *f*, NdhH, NdhS, PGR5, PGRL1, and PTOX. Appropriate amount of thylakoid extract (based on protein content) was separated with SDS‐PAGE and probed with specific antibodies. Equal loading was confirmed by protein staining with Li‐Cor Revert Total Protein Stain. (b) Relative content of proteins in mutant lines as percentage of WT. The numbers represent the average protein content ±*SE* in three to five biological replicates. The quantified values were normalized to the total protein content in the sample determined with Li‐Cor Revert Total Protein Stain. Statistically significant differences to WT according to Student's *t* tests (*p *<* *0.05) are marked with *

Differences in the rate of PQ reduction could also be caused by altered content of PSII or PSI complexes, the NDH complex, Cyt *b6f* or plastid terminal oxidase (PTOX). No statistically significant differences were detected in the amounts of the PSII core protein D1, Cyt *b6f* subunit Cyt *f*, or NDH subunits NhdS and NdhH between the studied lines, while a decrease in the amount of PGR5 and the PSI core protein PsaB, as well as elevated PTOX content were detected in *ntrc* (Figure 3).

### 2.3 NTRC promotes dark reduction of the plastoquinone pool

**Figure 4:**
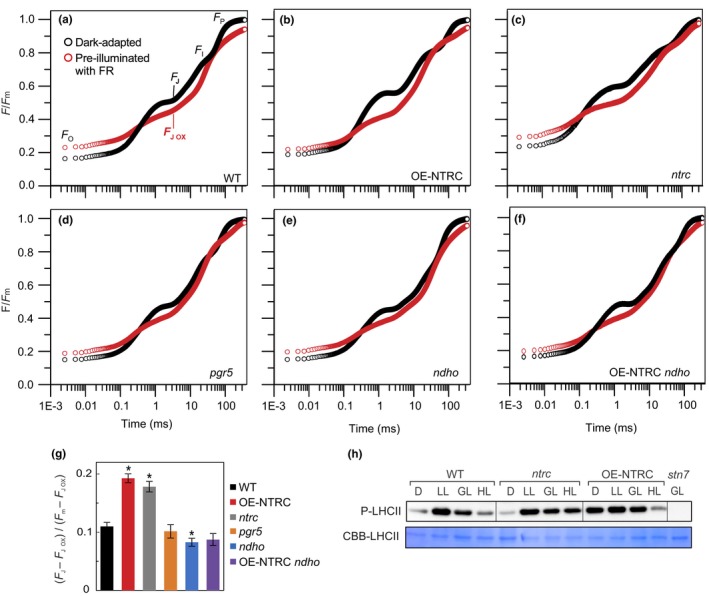
Redox state of the PQ pool in dark‐adapted leaves. (a–f) PQ pool redox state in darkness was determined from measurement of the OJIP transients in dark‐adapted leaves (black) and leaves preilluminated with far red light (red). Data are presented as averaged curves from measurements of 5 to 10 individual leaves on a logarithmic time scale from WT (a), OE‐NTRC (b), *ntrc* (c), *pgr5* (d), *ndho* (e), and OE‐NTRC *ndho* (f). The slight rise in F_0_ values after FR‐illumination is likely due to the small actinic effect of FR on PSII, as discussed by Schansker and Strasser (2005). (g) Proportions of reduced Q_A_ calculated as (F_J_‐F_J__ox_)/(F_m_‐F_J__ox_). Values are averages of measurements from 5 to 10 individual leaves ± *SE*. * indicates statistically significant difference to WT according to Student's *t* test (*p* < 0.05). All values are normalized to dark‐adapted Fm. (h) Determination of phosphorylation status of LHCII proteins in WT,* ntrc*, OE‐NTRC, and *stn7* after 2 hr of darkness and in low light (40 μmol photons m^−2^ s^−1^), growth light (200 μmol photons m^−2^ s^−1^) and high light (600 μmol photons m^−2^ s^−1^). Thylakoid extracts containing 0.4 μg chlorophyll were separated with SDS‐PAGE and phosphoproteins were detected with a Phosphothreonine‐specific antibody. Coomassie Brilliant Blue staining of LHCII on the membrane (CBB‐LHCII) was used as loading control

To determine whether the higher NDH activity that was observed in darkness after illumination of OE‐NTRC leaves (Figure 2a) alters the redox state of the PQ pool, we proceeded to analyze the phosphorylation level of LHCII proteins in dark‐adapted and illuminated leaves. The reduction in the PQ pool induces phosphorylation of LHCII by activating the STN7 kinase through interaction with the Cyt *b6f* complex (Bellafiore, Barneche, Peltier, & Rochaix, 2005; Shapiguzov et al., 2016; Vener, VanKan, Rich, Ohad, & Andersson, 1997). In WT plants LHCII proteins were mostly nonphosphorylated in darkness, maximally phosphorylated in low light, moderately phosphorylated in growth light, and mostly dephosphorylated in high light (Figure 4h), in agreement with earlier studies (Rintamäki et al., 1997; Tikkanen, Grieco, Kangasjarvi, & Aro, 2010). The *stn7* mutant was unable to phosphorylate LHCII (Bellafiore et al., 2005). In contrast to WT, LHCII was phosphorylated in darkness in OE‐NTRC (Figure 4h). As in WT, only a small amount of phosphorylated LHCII was present in thylakoids isolated from dark‐adapted leaves of *ntrc*, whereas slightly higher accumulation of phosphorylated LHCII was observed in high light‐illuminated *ntrc* (Figure 4h).

To further investigate the effect of NTRC on the reduction state of the PQ pool in darkness, we measured the kinetics of Chl a fluorescence OJIP transients in dark‐adapted leaves and in leaves preilluminated with far red light (FR) to fully oxidize the PQ pool. The difference in F/Fm at the J phase of the transient (F_J_, 3 ms after onset of illumination) between dark‐adapted and preilluminated leaves is an indicator for the redox state of the PQ pool in darkness (Stirbet, Riznichenko, Rubin, & Govindjee, 2014; Toth, Schansker, & Strasser, 2007). As demonstrated in Figure 4, the OE‐NTRC line had a significantly larger proportion of reduced PQ in darkness when compared to WT. The *ndho* mutant had a more oxidized PQ pool in darkness than WT, while there was no significant difference between *pgr5* and WT (Figure 4g), suggesting that the NDH complex is the main CEF pathway contributing to dark‐reduction of the PQ pool. The differences in dark‐redox state of PQ (Figure 4g) were also supported by an elevated dark‐adapted apparent F_O_ value, a steeper initial slope of the transient, and a decreased area above the O‐J phase of the OJIP transient in both *ntrc* and OE‐NTRC when compared to WT (Supporting Information Table S1) (Toth et al., 2007). Attribution of the elevated dark‐reduction of PQ in OE‐NTRC to enhanced activity of the NDH complex was further supported by the observation that the redox state of the PQ pool in darkness was more oxidized in the OE‐NTRC *ndho* line in comparison to WT and OE‐NTRC (Figure 4g). OJIP transients also showed higher dark‐reduction of PQ in *ntrc* mutant when compared to WT (Figure 4), but it must be noted that the overall kinetics of the OJIP transient in *ntrc* differed considerably from the other lines.

### 2.4 NTRC enhances the generation of proton motive force during dark‐to‐light transitions

Reduction of plastoquinone by the thylakoid NDH complex is known to be coupled with the translocation of protons to the lumen, which contributes to the formation of *pmf* and enhances ATP synthesis (Strand, Fisher, & Kramer, 2017). We therefore investigated whether the generation of *pmf* during dark‐to‐light transitions and in plants illuminated with different light conditions is affected by deficiency or overexpression of NTRC. The *pmf*, conductivity of the thylakoid membrane to protons (*g*_H+_), and proportions of the *pmf* components ΔpH and ΔΨ were determined by measuring a difference in absorbance at 550 and 515 nm, also known as the electrochromic shift (ECS) (Cruz et al., 2005).

**Figure 5:**
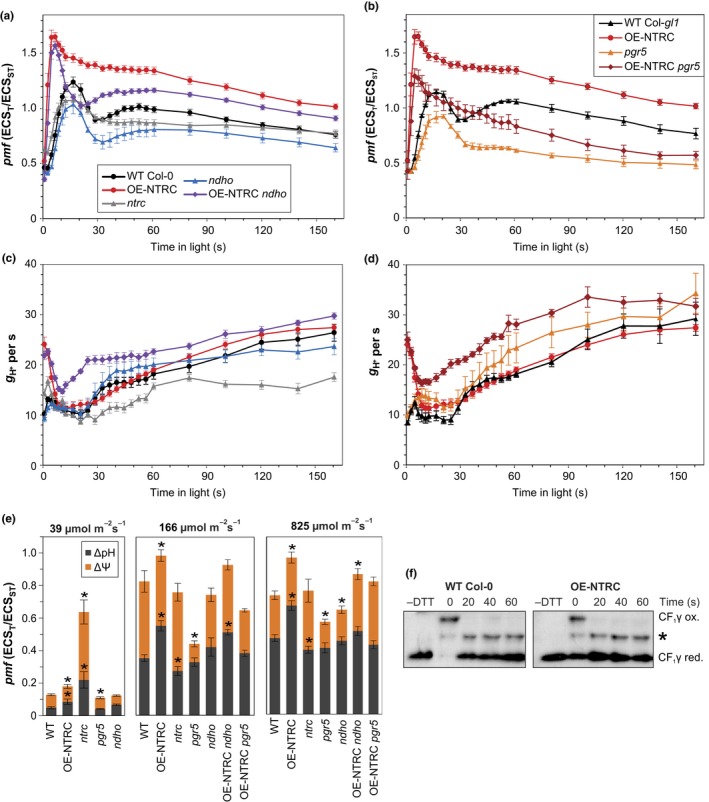
Generation of the proton gradient during dark‐to‐light transitions. (a, b) Proton motive force (*pmf*) at specific time points during transitions from dark to actinic light of 166 μmol photons m^−2^ s^−1^ (AL) in dark‐adapted leaves of WT Col‐0, OE‐NTRC,* ntrc*,* ndho,* and OE‐NTRC *ndho* (a) and in WT Col‐*gl1*, OE‐NTRC,* pgr5,* and OE‐NTRC *pgr5* (b). The *pmf* was measured as light‐induced change in the ECS signal (ECS_T_) and normalized with the magnitude of ECS induced by a 20‐μs saturating single‐turnover flash administered prior to the onset of AL (ECS_ST_). Values are averages of measurements from 4 to 16 individual leaves ±*SE*. (c, d) Conductivity of the thylakoid membrane to protons (*g*_H__+_), calculated as the inverse of the time constant of a first‐order fit to the decay of ECS during 250 ms dark intervals. (e) Total *pmf* and its partitioning to ΔpH and ΔΨ after 3 min illumination with low, growth or high light. Values are averages of measurements from 3 to 6 (for 39 μmol m^−2^ s^−1^), 5 to 10 (for 166 μmol m^−2^ s^−1^), and 8 to 15 (for 825 μmol m^−2^ s^−1^) individual leaves ±*SE*. Statistically significant differences to WT according to Student's *t* tests are marked with *. (f) Mobility shift assays with MAL‐PEG labeled protein extracts to determine the in vivo redox state of CF_1_γ subunit of the ATP synthase during first 60 s of dark‐to‐growth light transitions. * marks an unspecific band of unknown origin

**Figure 6:**
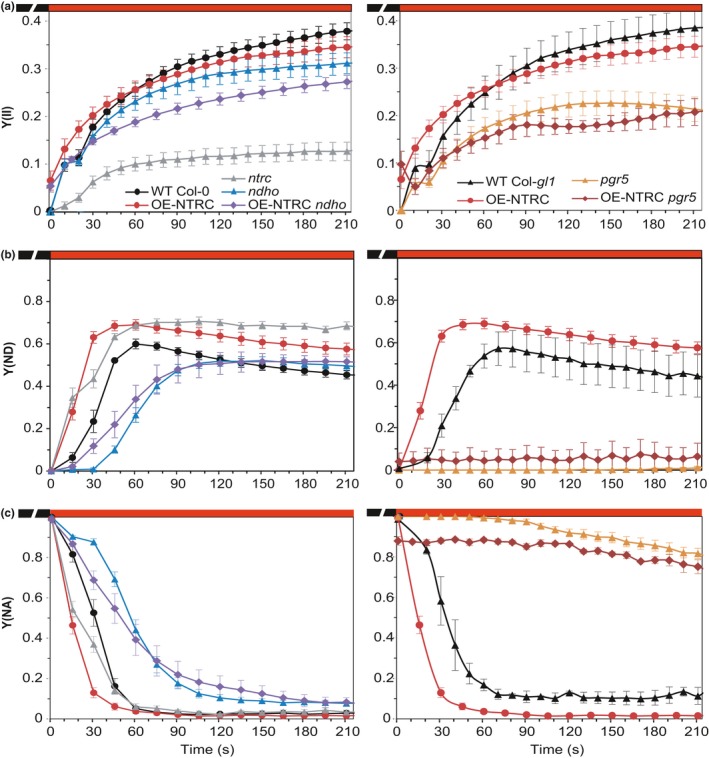
Photosynthetic parameters measured from leaves during dark‐to‐light transitions. (a–c) Dark‐adapted WT Col‐0, *ntrc*, OE‐NTRC,* ndho*, OE‐NTRC *ndho*, WT Col‐*gl1*,* pgr5*, and OE‐NTRC *pgr5* leaves were shifted from darkness to the actinic light of 166 μmol photons m^−2^ s^−1^ and illuminated for 210 s. Photosystem II quantum yield (a), was calculated from Chl a fluorescence. P700 oxidation (Y(ND)) (b) and PSI acceptor side limitation (Y(NA)) (c) were calculated from absorbance difference at 875 and 830 nm. The graphs are averages of measurements from four to nine individual leaves ±*SE*

In WT, *pmf* was transiently elevated upon onset of illumination at growth light intensity, peaking after 15–20 s (Figure 5a) and coinciding with a decrease in *g*_H+_ (Figure 5c). The initial decrease in *g*_H+_ occurred despite rapid reduction of the gamma subunit of the ATP synthase (CF_1_γ), as after 20 s under growth light CF_1_γ was already fully reduced (Figure 5f). Under growth light intensity, another slight rise in *pmf* was observed after ca. 30–60 s in light (Figure 5a), coinciding with P700 oxidation (Figure 6b). Subsequently *pmf* slowly decreased to a steady‐state value.

In OE‐NTRC, the initial *pmf* increase occurred already in a few seconds after the onset of illumination, reached a higher level and decreased more slowly than in WT (Figure 5a). While *g*_H+_ was drastically elevated in dark‐adapted OE‐NTRC leaves, it rapidly decreased to a level comparable to WT (Figure 5c). Both total *pmf* and the contribution of ΔpH to it were higher in OE‐NTRC in all light intensities when compared to WT (Figure 5e). There was no significant difference between OE‐NTRC and WT in *g*_H+_ under growth light illumination apart from the enhanced conductivity in dark‐adapted OE‐NTRC leaves (Figure 5c). PSII quantum yield (Figure 6a) increased only slightly during early photosynthetic induction and slightly but insignificantly decreased at steady‐state illumination in comparison to WT, while P700 oxidation was significantly faster (Figure 6b). These results suggested that the increase in *pmf* in OE‐NTRC derives from CEF, which is also supported by the nonlinear relationship between thylakoid proton flux (*v*_H+_) and quantum yield of PSII in plants with modified NTRC content (Supporting Information Figure S3).

In *ndho*, generation of *pmf* followed similar kinetics as in WT, but its level remained lower (Figure 5a). In comparison to WT, thylakoid proton conductivity was increased during the 30–60‐s time period (Figure 5c). In OE‐NTRC *ndho*, however, an elevation of *pmf* occurred during transitions from dark to growth light similarly to OE‐NTRC, except for a time period between 15–40 s after onset of illumination, where *pmf* was lowered in OE‐NTRC *ndho* in comparison to OE‐NTRC (Figure 5a). Upon dark‐to‐light transitions, the high initial thylakoid proton conductivity in OE‐NTRC *ndho* decreased as in OE‐NTRC, but after 10 s of illumination *g*_H+_ again rose more rapidly than in OE‐NTRC (Figure 5c). In the absence of the NDH complex, NTRC overexpression was also unable to enhance P700 oxidation during dark‐to‐growth light transitions (Figure 6b), and OE‐NTRC *ndho* suffered from increased PSI acceptor side limitation in comparison to WT (Figure 6c). These data indicate that the enhanced capacity of the stroma in OE‐NTRC to pull electrons from PSI during dark‐to‐light transitions is dependent on the NDH complex.

Increased activation of the NDH complex is not, however, sufficient to fully explain the elevated *pmf* in OE‐NTRC, especially immediately after dark‐to‐light transitions and at steady state. Therefore, we also generated an NTRC overexpression line in the *pgr5* mutant background (OE‐NTRC *pgr5*) whose NTRC expression level was comparable to OE‐NTRC (Supporting Information Figure S1B). In the *pgr5* mutant, *pmf* generation at the onset of illumination was impaired and the steady‐state level of *pmf* was lower than in WT (Figure 5b), in part due to increased *g*_H+_ (Figure 5d). The absence of PGR5 did not alter the kinetics of *pmf* induction in plants overexpressing NTRC, but the magnitude of *pmf* remained lower (Figure 5b). During early photosynthetic induction, proton conductivity of the thylakoid membrane in OE‐NTRC *pgr5* was high, like in OE‐NTRC, but dropped less than in OE‐NTRC, and at steady state, stayed higher even in comparison to *pgr5* (Figure 5d). Moreover, PSI acceptor side limitation was only slightly alleviated in OE‐NTRC *pgr5* in comparison to *pgr5* (Figure 6c). Interestingly, a high initial *g*_H+_ value and rapid generation of a *pmf* peak after onset of illumination were observed in all plants overexpressing NTRC (OE‐NTRC, OE‐NTRC *ndho*, and OE‐NTRC *pgr5*) but missing in WT, *ndho*, and *pgr5* plants (Figure 5a,b).

In *ntrc*, the initial *pmf* increase occurred with similar kinetics to WT upon onset of illumination at growth light intensity, but had a lesser magnitude (Figure 5a). The secondary increase in *pmf* was absent in *ntrc* leaves. In growth light, the steady‐state *pmf* was comparable to WT, but contribution of ΔpH to total *pmf* was slightly diminished (Figure 5e). Decreased thylakoid conductivity to protons was observed in growth light intensity in *ntrc* (Figure 5c). However, CF_1_γ is reduced normally in growth light in *ntrc* (Nikkanen et al., 2016), implying that thylakoid proton conductivity is inhibited by other means. A high donor side limitation of PSI was measured in *ntrc* under growth light (Figure 6b) despite high excitation pressure in the PQ pool (Supporting Information Figure S4B) and lowered PsaB content (Figure 3), suggesting inhibition of electron transfer between the PQ pool and PSI.

### 2.5 In fluctuating light, NTRC overexpression enhances PSI yield in low light and represses thylakoid conductivity to protons upon transitions from low to high irradiance

**Figure 7:**
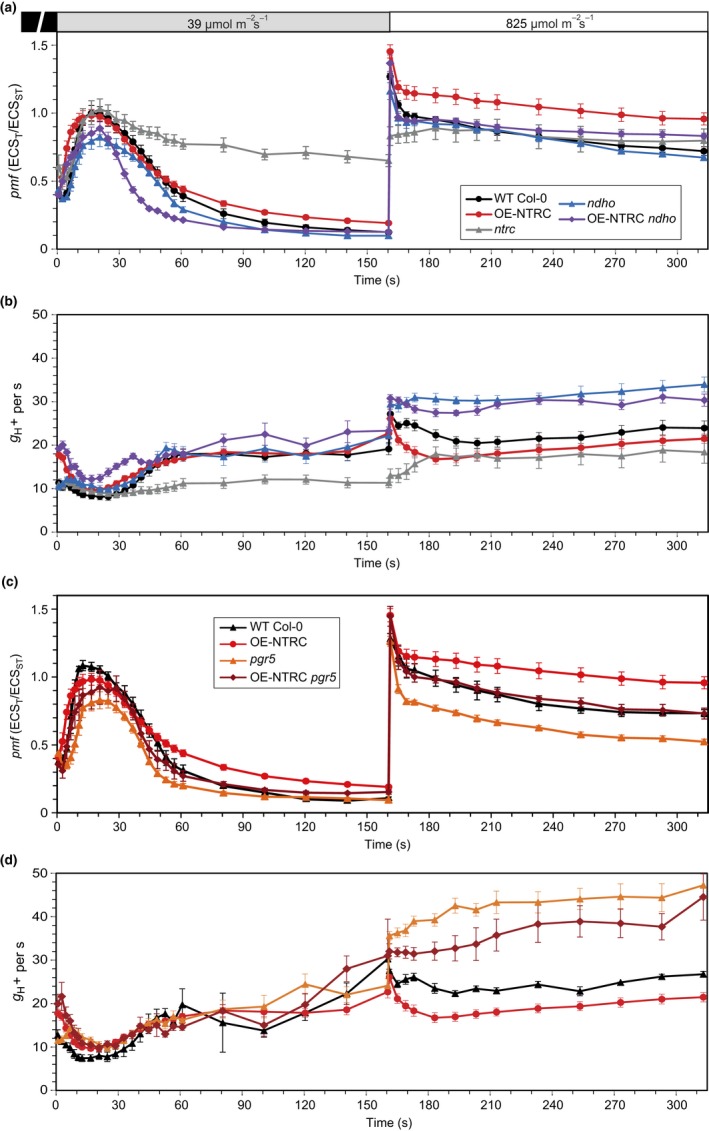
Formation and regulation of the proton motive force during changes in light conditions. (a, b) The *pmf* (a) and proton conductivity of the thylakoid membrane (*g*_H__+_) (b) at specific time points during transitions from darkness to low actinic light (39 μmol photons m^−2^ s^−1^) and from low to high light (825 μmol photons m^−2^ s^−1^) in dark‐adapted leaves of WT Col‐0, OE‐NTRC,* ntrc*,* ndho,* and OE‐NTRC *ndho*. The *pmf* and *g*_H__+_ were measured and calculated as explained in the legend for Figure 5. The graphs shown are averages of measurements from 4 to13 individual leaves ±*SE*. (c, d) The *pmf* (c) and *g*_H__+_ (d) in dark‐adapted leaves of WT Col‐*gl1*, OE‐NTRC,* pgr5,* and OE‐NTRC *pgr5* during changes in light conditions. The experiment was performed as explained in the figure legend for (a, b). The graphs shown are averages of measurements from 3 to 13 individual leaves ±*SE*

**Figure 8:**
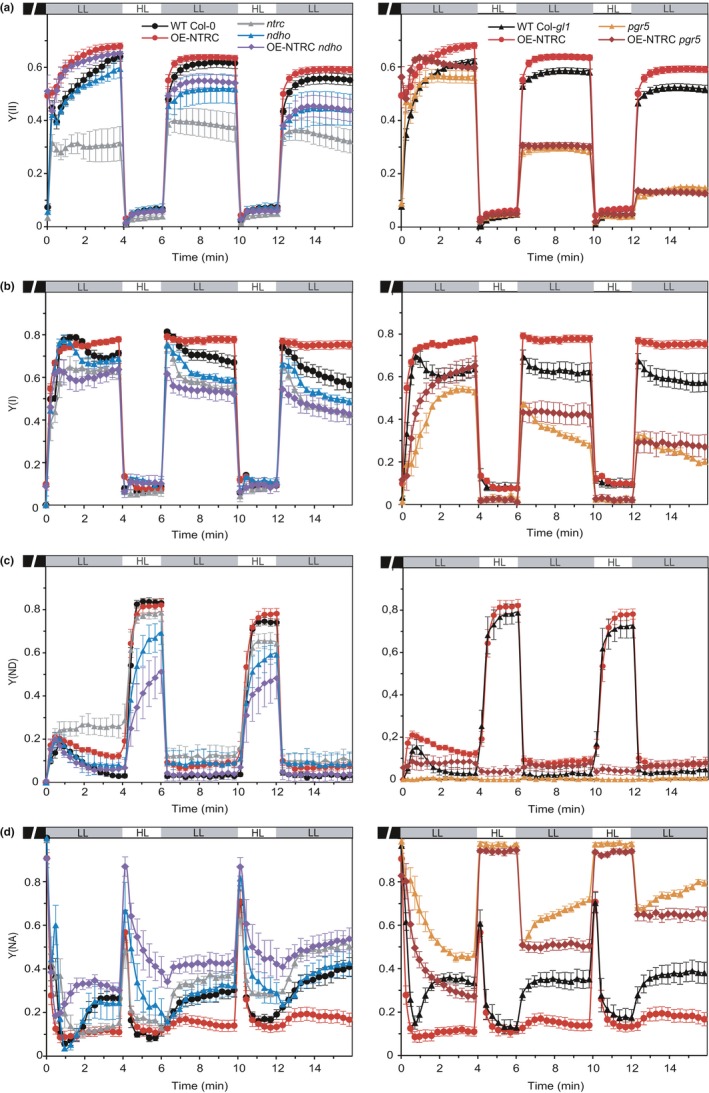
Analysis of Chlorophyll a fluorescence and P700 oxidation in fluctuating light. (a) PSII yield (Y(II)) in light conditions fluctuating between periods of low actinic light (LL, 39 μmol photons m^−2^ s^−1^) and high light (HL, 825 μmol photons m^−2^ s^−1^) in WT Col‐0, OE‐NTRC,* ntrc*,* ndho*, OE‐NTRC *ndho*, WT Col‐*gl1*,* pgr5,* and OE‐NTRC *pgr5*. Five‐week‐old plants were dark‐adapted for 30 min before measuring fluorescence from detached leaves. All values are averages of measurements from 3 to 10 individual leaves ±*SE*. (b–d) PSI yield (Y(I)) (b), P700 oxidation (Y(ND)) (c), and PSI acceptor side limitation (Y(NA)) (d) in light conditions fluctuating between periods of low actinic light (LL, 39 μmol photons m^−2^ s^−1^) and high light (HL, 825 μmol photons m^−2^ s^−1^) in WT Col‐0, OE‐NTRC,* ntrc*,* ndho*, OE‐NTRC *ndho*, WT Col‐*gl1*,* pgr5,* and OE‐NTRC *pgr5*. Five‐week‐old plants were dark‐adapted for 30 min before measuring fluorescence from detached leaves. All values are averages of measurements from 3 to 10 individual leaves ±*SE*

Due to the recent suggestions implicating a particularly important role for NTRC under low and fluctuating light conditions (Carrillo et al., 2016; Nikkanen et al., 2016; Thormählen et al., 2017), we proceeded to investigate *pmf* generation and photosynthetic electron transfer in OE‐NTRC under these conditions. During transitions from dark to low light intensity, increased *pmf* formation was again observed in OE‐NTRC, but the difference to WT was less dramatic than in growth light (Figure 7a). PSII yield was enhanced in OE‐NTRC during the transition from dark to low light (Figure 8a), also contributing to the *pmf* increase. P700 oxidation was also enhanced during dark‐to‐low light transitions (Figure 8c), while NPQ was decreased (Supporting Information Figure S5A), despite higher ΔpH (Figure 5e). A high PSI yield was maintained throughout the low light periods in OE‐NTRC due to low acceptor side limitation (Figure 8b). Notably, the overexpression of NTRC in *ndho* background reverted these changes observed in OE‐NTRC plants to levels comparable to or even more severe than in *ndho* knockout plants, except for the increased PSII yield during dark‐to‐low light transitions (Figures 7a,b and 8a), suggesting that enhanced activation of NDH‐mediated CEF contributes to photosynthetic performance of OE‐NTRC during dark‐to‐light transitions and under low light.

During transitions from dark to low light, the *pgr5* and *ndho* mutants both showed impaired *pmf* generation (Figure 7). In contrast, *pmf* generation in OE‐NTRC *pgr5* during dark‐to‐low and low‐to‐high light transitions was recovered to WT levels (Figure 7c) despite elevated *g*_H+_ especially in high light (Figure 7d). The recovery of *pmf* most likely occurred due to enhanced activity of NDH‐CEF by increased NTRC content. Slightly improved tolerance to light fluctuation was also observed in OE‐NTRC *pgr5* in comparison to *pgr5* as PSI yield was better maintained in low light following the high light periods (Figure 8b). Importantly, overexpression of NTRC improved the ability to oxidize PSI in low light even in the *pgr5* background (Figure 8c).

In all lines a transient *pmf* spike was observed upon the switch from low to high light intensity (Figure 7). In WT, the proton conductivity of the thylakoid membrane decreased gradually (Figure 7b), but the decrease in *g*_H_+ was not due to oxidation of CF_1_γ, as it remained fully reduced in high light conditions (Supporting Information Figure S6B). The decrease in *g*_H+_ was even stronger in OE‐NTRC upon the shift from low to high light (Figure 7b). Overexpression of NTRC in *ndho* background decreased *pmf* generation during transitions from low to high light in comparison to the OE‐NTRC line (Figure 7a), suggesting that enhanced activation of NDH‐mediated CEF contributes to the high *pmf* in OE‐NTRC in these conditions. OE‐NTRC *ndho* showed increased steady‐state *g*_H+_ under high irradiance similarly to *ndho* (Figure 7b).

The *pgr5* mutant was unable to oxidize P700 in high light (Figure 8c) or to decrease proton efflux from the lumen (Figure 7d), resulting in a loss of *pmf* (Figure 7c). The strong decrease in *g*_H+_ observed in OE‐NTRC in high light disappeared in OE‐NTRC *pgr5,* which lacks PGR5 (Figure 7d).

In *ntrc*, high steady‐state *pmf* under low light intensity (Figures 5e, and 7a) was likely caused by impaired activation of the chloroplast ATP synthase and the Calvin–Benson cycle as previously reported (Carrillo et al., 2016; Nikkanen et al., 2016). Furthermore, exceptionally high NPQ was recorded in the *ntrc* line, especially at low light (Supporting Information Figure S5A).

Concluding from Figures 5, 6, 7, 8, it is evident that both the knockout and overexpression of NTRC had a distinct influence on the formation of *pmf* during transitions from dark to light and from low to high light through regulation of the activities of CEF and ATP synthase.

### 2.6 Interaction of CEF‐related proteins with NTRC

Distinct effects of NTRC overexpression or deficiency on the postillumination fluorescence rise (Figure 2), the dark‐reduction level of the PQ pool (Figure 4), and generation of *pmf* during dark/light transitions and low/high light transitions (Figures 5 and 7) suggested that NTRC may either directly or indirectly regulate CEF. In order to screen for potential targets of NTRC‐mediated regulation, we performed coimmunoprecipitation (Co‐IP) assays with an antibody against NTRC, and analyzed eluates from WT, *ntrc*, and OE‐NTRC total leaf extracts by mass spectrometry (MS). A full list of identified peptides is provided in Supporting Information Dataset S1, while Supporting Information Table S2 lists 100 chloroplast proteins in order of their abundance in WT and OE‐NTRC eluates but missing in *ntrc* eluates. Among these proteins were several TRX interactors identified in previous studies (Balmer et al., 2003; Marchand et al., 2006; Hall et al., 2010), as well as established NTRC target proteins such as 2‐cysteine peroxiredoxins (Pérez‐Ruiz et al., 2006) and enzymes involved in chlorophyll biosynthesis (Richter et al., 2013). Relevantly in the current context, several proteins involved in CEF around PSI were identified by the Co‐IP/MS screening (Table 1). Most notably, five subunits of the thylakoid NDH complex, NdhH, Ndh48, NdhS, NdhU, and NdhO, (in order of abundance in the Co‐IP eluates) as well as PGR5 were identified as potential NTRC interactors. Intriguingly, all of the NDH subunits identified are located in close proximity to the proposed ferredoxin binding and oxidation site on the stromal side of the NDH complex (Peltier et al., 2016; Shikanai, 2016; Yamamoto & Shikanai, 2013; Yamamoto et al., 2011).

**Table 1:** Screening of putative NTRC target proteins in CEF pathways by Co‐IP/MS

AGI code	Description	*ntrc*	WT	OE‐NTRC	MW (kDa)	#Cys
ATCG01110.1	NdhH	‐	+	+	45.5	3
AT1G15980.1	Ndh48 (NDF1)	‐	+	+	51.0	3
AT4G23890.1	NdhS (CRR31)	‐	+	+	27.7	2
AT1G74880.1	NdhO	‐	+	+	17.6	0
AT5G21430.2	NdhU	‐	+	‐	24.3	0
AT2G05620.1	PGR5	‐	+	‐	14.3	1

*Notes*

Only proteins of which at least two unique peptides were detected in WT/OE‐NTRC eluates (+), and which were absent from *ntrc* eluates are included in the table. MW (kDa) indicates molecular weight of the protein, and #Cys the number of conserved cysteine residues (see Supporting Information Tables S3–S5). For a description of experimental procedures see Materials and Methods. For a list of 100 chloroplast proteins detected in WT and/or OE‐NTRC but not in *ntrc* eluates, see Supporting Information Table 2, and for a full list of detected peptides see Supporting Information Dataset S1.

**Figure 9:**
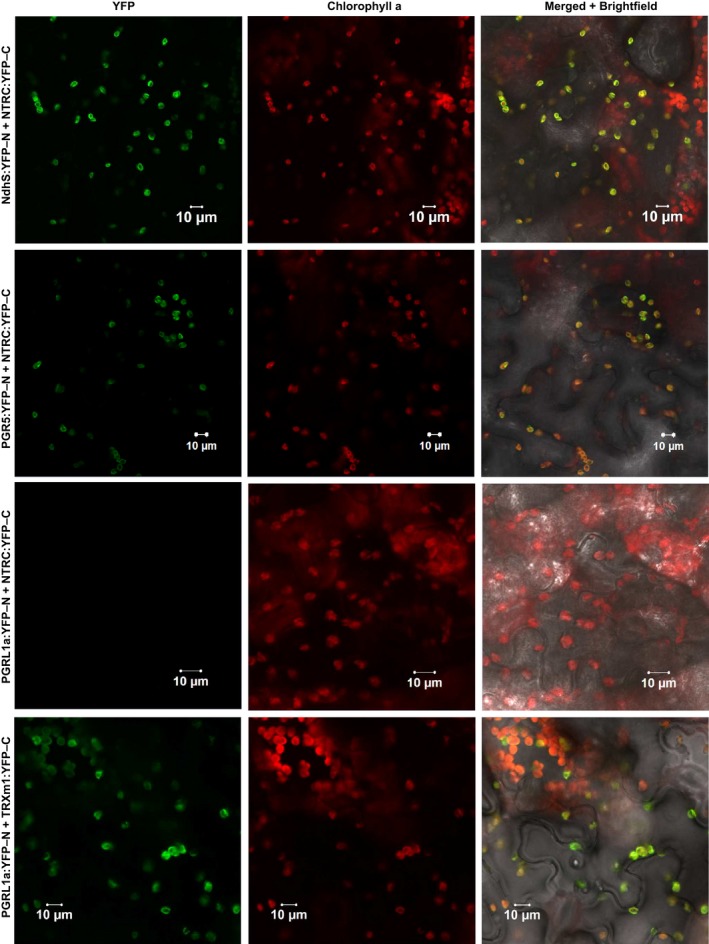
Bimolecular fluorescence complementation (BiFC) tests of *in planta* interactions between chloroplast TRXs and potential target proteins in cyclic electron flow. The left panel shows yellow fluorescent protein (YFP) fluorescence in green, the middle panel Chlorophyll a autofluorescence in red and the right panel, a merged image of YFP, chlorophyll, and brightfield images. YFP‐N and YFP‐C indicate expression of a fusion proteins including the N‐terminal and C‐terminal parts of YFP, respectively, in tobacco (*Nicotiana benthamiana*) leaves

The potential interactions of NTRC with NdhS and PGR5 were further supported by positive results in bimolecular fluorescence complementation tests (BiFC). Coexpression of NTRC with both NdhS and PGR5 in *Nicotiana benthamiana* leaves resulted in YFP fluorescence that was strictly colocalized with chlorophyll autofluorescence, suggesting that it originated from the thylakoid membrane (Figure 9). TRX‐*m*1 interacted with PGRL1, while no interaction capability was detected between PGRL1 and NTRC (Figure 9). Neither NdhS nor PGR5 interacted with TRX‐*x* in BiFC, which was used as a control in the test (Supporting Information Figure S7).

To assess the potential of the CEF‐related proteins identified by Co‐IP/MS (Table 1) and BiFC (Figure 9) to be targets of redox regulation by TRXs, their amino acid sequences were analyzed for conserved cysteine residues. Of the NDH subunits identified as putative NTRC targets by Co‐IP/MS, NdhS, NdhH, and Ndh48 contain cysteine residues that are conserved in angiosperms (Table 1, Supporting Information Tables S3–S5), and therefore could in theory be subject to thiol regulation. NdhO and NdhU do not contain conserved cysteine residues, but they likely coprecipitate with the NTRC antibody because of their interactions with NdhH and NdhS subunits of the NDH complex, respectively (Shikanai, 2016).

PGR5 has been shown to form redox‐dependent heterodimers with PGRL1 which have been proposed to be required for acceptance of electrons from ferredoxin and for reduction of PGRL1 (Hertle et al., 2013; Leister & Shikanai, 2013). The mature PGR5 polypeptide contains a single highly conserved cysteine residue (Munekage et al., 2002), which could hypothetically form an intermolecular disulfide with PGRL1 or some other partner, or be a target for S‐nitrosylation or glutathionylation (Couturier, Chibani, Jacquot, & Rouhier, 2013; Zaffagnini et al., 2016). As a direct determination of PGR5 redox state with the alkylation method was not feasible, we investigated if the redox state of PGRL1 is affected by NTRC deficiency or overexpression. PGRL1 contains six conserved thiol‐sensitive cysteine residues that form inter‐ and intramolecular disulfides (Hertle et al., 2013; Petroutsos et al., 2009). We observed that PGRL1 was mostly oxidized in dark‐adapted leaves, but underwent a transient reduction during approximately 60 s of illumination with growth light (Supporting Information Figure S6A). This corresponds with the timescale of NPQ induction (Supporting Information Figure S5A), as well as with the transient increase in *pmf* and decrease in *g*_H+_ during dark‐to‐light transitions (Figure 5a–d). No significant difference to WT in PGRL1 reduction or protein content was detected in OE‐NTRC (Supporting Information Figure S6A, Figure 3). PGR5 content of thylakoid membranes was, however, decreased in *ntrc* by 40% in comparison to WT (Figure 3).

## 3 DISCUSSION

The role of CEF around PSI in the response of plants to fluctuating light conditions has attracted great attention during the past 10 years. Importance of CEF likely relies in its capacity to maintain redox balance in chloroplasts upon fluctuations in light intensity and during dark‐to‐light transitions (Strand, Fisher, & Kramer, 2016; Suorsa et al., 2016; Yamori et al., 2016). Plastidial thioredoxin systems, on the other hand, are crucial regulators of chloroplast metabolic reactions in the stroma. It has, however, remained unclear whether thioredoxin‐related redox regulation is also involved in the attainment of CEF‐mediated redox balance upon exposure of plants to changing light intensities. To this end, we applied here an *in vivo* approach to investigate, whether NTRC contributes to regulation of CEF pathways in chloroplasts.

### 3.1 Regulation of photosynthesis is altered in the pleiotropic *ntrc* mutant

While the *ntrc* knockout plants are plain green and stunted (Lepistö et al., 2009; Pérez‐Ruiz et al., 2006; Serrato et al., 2004), the rosettes of OE‐NTRC lines are healthy and vigorous (Nikkanen et al., 2016; Toivola et al., 2013). Accordingly, the photosynthetic parameters measured for the *ntrc* knockout plants were not always in line with the results obtained with NTRC‐overexpressing plants (Figures 2 and 4). In a short photoperiod, the *ntrc* plants have a highly pleiotropic phenotype (Supporting Information Figure S2A,B) (Kirchsteiger et al., 2009; Naranjo et al., 2016; Nikkanen et al., 2016; Pérez‐Ruiz et al., 2006 Pulido et al., 2010; Thormählen et al., 2015) that complicates the interpretation of results from the *ntrc* line. The high, slow‐rising PIFR and increased dark‐reduction of PQ in short‐day grown *ntrc* (Figures 2a and 4g) were likely caused by a high NPQ (Supporting Information Figures S2B and S5A) and its relaxation in darkness, lowered PSI content (Figure 3) (Thormählen et al., 2015), and impaired stromal redox metabolism (Nikkanen et al., 2016; Pérez‐Ruiz et al., 2006). Furthermore, we observed increased accumulation of reduced Fd, the substrate of the NDH complex, in *ntrc* (Figure 2d), which may be due to lower activity of CBC enzymes and consequent lower consumption of NADPH in carbon fixation (Nikkanen et al., 2016). Accordingly, a high NADPH/NADP^+^ ratio has been shown to activate CEF (Breyton et al., 2006; Okegawa, Kagawa, Kobayashi, & Shikanai, 2008). A high NADPH/NADP+ ratio together with high accumulation of reduced Fd may also explain the activation of NDH‐dependent CEF by H_2_O_2_ (Strand et al., 2015; Strand, Fisher, & Kramer, 2017; Strand, Livingston, et al., 2017), since oxidative treatment decreases the activation of redox‐regulated CBC enzymes, and consequently the consumption of NADPH in illuminated chloroplasts. Accordingly, the *ntrc* mutant has a higher accumulation of H_2_O_2_ than WT (Supporting Information Figure S2C and Pulido et al., 2010), a higher NADPH/NADP+ ratio (Thormählen et al., 2015), and lower activation states of CBC enzymes (Nikkanen et al., 2016; Pérez‐Ruiz et al., 2006), which likely all contribute to the altered PIFR observed in Figure 2a. The PIFR was significantly diminished in *ntrc* plants grown in a longer photoperiod, where NPQ is lower (Supporting Information Figure S2A) and the phenotype of *ntrc* has been shown to be less severe in terms of growth, chlorophyll content, and efficiency of photochemistry (Lepistö et al., 2009, 2013). In *ntrc*, electrons also accumulate in the PQ pool upon increases in light intensity (Supporting Information Figure S5), although PSI is limited on the donor side (Figures 6 and 8), indicating that electron transfer is limited between the PQ pool and PSI. Higher content of PTOX (Figure 3) may assist relaxation of excitation pressure in the PQ pool of *ntrc* plants.

The pleiotropy described above for *ntrc* complicates the general interpretation of the results and makes it difficult to assess the contribution of NTRC to the regulation of photosynthesis when using the *ntrc* line alone in the experiments. To avoid these obstacles we have constructed the OE‐NTRC line, whose phenotype and development are not considerably dissimilar to WT (Nikkanen et al., 2016; Toivola et al., 2013). OE‐NTRC in different backgrounds (*ntrc*,* ndho* and *pgr5*) provides a more reliable platform to examine the direct effects of NTRC on specific plastidial processes.

### 3.2 NTRC in regulation of CEF

In‐depth analysis of NTRC overexpression lines with respect to thylakoid functional properties provided evidence that NTRC is indeed involved in regulation of CEF in the thylakoid membrane. Support for NTRC‐induced activation of the NDH complex was obtained by analyzing thylakoid CEF‐related functions in NTRC overexpression lines made on the backgrounds of *ndho* (OE‐NTRC *ndho*) and *pgr5* (OE‐NTRC *pgr5*), incapable of performing NDH‐ and PGR‐dependent CEF, respectively. A distinct effect of NTRC overexpression on the postillumination rise of chlorophyll a fluorescence (Figure 2)*,* the redox state of the plastoquinone pool in darkness (Figure 4) as well as on the generation of the *pmf* (Figures 5 and 7) and oxidation of P700 (Figures 6b and 8c) upon dark‐to‐light transitions and sudden increases in light intensity demonstrated the activating effect of NTRC on NDH‐dependent CEF. Higher activity of NDH‐dependent CEF in plants overexpressing NTRC was shown not to be due to increased accumulation of the NDH complex or its substrate, because no increase in the accumulation of either NDH subunits or reduced Fd was detected in OE‐NTRC plants in comparison to WT (Figures 2d and 3). Furthermore, identification of NDH subunits in close proximity of the ferredoxin binding site as potential NTRC interactors supports the proposition that NTRC may have a direct effect on the activation of NDH (Table 1, Figure 9). Although several NDH subunits were detected by Co‐IP/MS, most likely only one or few of these subunits are genuine NTRC targets. The others likely coprecipitate with the NTRC antibody due to reciprocal interactions of the NDH subunits on the stromal side of the thylakoid membrane (Peltier et al., 2016; Shikanai, 2016). Existence of a thiol‐regulated component in the ferredoxin binding site would provide a mechanism for dynamic control of the ferredoxin:plastoquinone oxidoreductase activity of the complex in response to fluctuations in light conditions. Redox regulation of the NDH complex would allow rapid adjustment of *pmf* and nonphotochemical quenching as well as the maintenance of a redox balance between the electron transfer chain and the electron sink capacity of stromal acceptors, most importantly the CBC. In high light, less active NDH could prevent the reverse function of the complex (i.e., oxidization of PQ to reduce ferredoxin and transfer of protons from lumen to stroma) in conditions of high *pmf* and a reduced PQ pool (Strand, Fisher, & Kramer, 2017).

Notably, overexpression of NTRC also affects the function of PGR5. Nevertheless, as discussed below, the effect is not necessarily related to the putative role of PGR5 in CEF. Our results, more likely, support the hypothesis (Kanazawa et al., 2017; Tikkanen, Rantala, & Aro, 2015) that PGR5 is involved in controlling the proton conductivity of the thylakoid membrane, which, consequently, affects the generation of *pmf*.

### 3.3 Regulation of *pmf* and the thylakoid redox balance via NDH and NTRC during changes in light conditions

Overexpression of NTRC caused elevated *pmf* under all light conditions, while no significant changes were observed in PSII quantum yield or thylakoid proton conductivity in comparison to WT (Figures 5, 6a, 7 and 8a). These results suggest that the elevation of *pmf* derives from enhanced CEF. Increased P700 oxidation during dark‐to‐light transitions in OE‐NTRC was reverted in OE‐NTRC *ndho* (Figure 6b), and a lack of NDH also delayed the ability to oxidize P700 during the high‐light phases in fluctuating light (Figure 8c). It is therefore evident that the NDH complex controls the strength of the trans‐thylakoid *pmf* as well as the redox balance between the electron transfer chain and the stroma, and that this regulation is under the control of the stromal TRX systems, with our results suggesting a specific role for the NTRC system.

While our results demonstrate enhancement of the NDH‐dependent CEF by NTRC overexpression (Figure 2), earlier studies have revealed an inhibition of NDH‐dependent CEF upon TRX‐*m*4 overexpression and, conversely, an enhancement in *trxm4* mutants (Courteille et al., 2013). Thus, it is conceivable that the two chloroplast TRX systems regulate CEF in an antagonistic way, although it remains to be elucidated how such a regulation might be mechanistically accomplished. We propose that in low light and upon sudden changes in the light intensity, NTRC is crucial for activation of the NDH‐dependent CEF, while the TRX‐*m4*‐dependent inhibition of NDH‐CEF requires higher light intensity or longer duration of illumination. Moderate to high light illumination is required to fully activate the FTR‐dependent TRX system (reviewed in Geigenberger et al., 2017), which possibly contributes to the downregulation of NDH. In OE‐NTRC, the NDH‐dependent CEF is constitutively active in light, which contributes to elevated *pmf* in all light intensities. Upon transition from dark to low light, there is less difference between OE‐NTRC and WT in terms of *pmf* formation (Figure 7a), because in those conditions the NTRC‐mediated activation of NDH occurs similarly in WT and OE‐NTRC.

The NDH complex translocates protons from the stroma to the lumen not only via Cyt *b6f* but also itself functions as a proton pump with a 2 H^+^/e^−^ stoichiometry (Strand, Fisher, & Kramer, 2017). NDH‐mediated CEF therefore contributes relatively more to *pmf* generation and consequently to ATP synthesis and NPQ induction than the PGR‐dependent pathway. It has been postulated that the NDH complex is unlikely responsible for CEF during the early induction phase of photosynthesis, due to a low concentration of the complex in thylakoids in relation to the total PSI content (Joliot & Joliot, 2002). However, the NDH complex forms functional CEF‐supercomplexes with PSI in stroma thylakoids (Peng et al., 2008) and recently it has been shown that a single NDH complex can bind up to six PSI complexes (Yadav et al., 2017). Specific association of NDH complex with several PSI complexes indicate that even a relatively low NDH content may have a significant impact on *pmf* generation. Moreover, in vivo results in the current paper as well as in another recent study (Shimakawa & Miyake, 2018) demonstrate the importance of the NDH complex for the redox poise of the photosynthetic electron transfer chain during dark‐to‐light transitions as well as during fast fluctuations in light intensity.

### 3.4 Contribution of TRX systems to PGR‐related CEF pathway

Increased activation of NDH‐CEF alone is not sufficient to explain all observed changes of *pmf* in OE‐NTRC plants. When compared to WT, *pmf* remained elevated in OE‐NTRC *ndho* during the first seconds of photosynthetic induction and at steady state in growth and high light (Figures 5a and 7a). These results could be explained by activation of PGR‐dependent CEF as well in plants overexpressing NTRC. Stromal thiol redox state has been previously suggested to control PGR‐dependent CEF by a component that has a midpoint redox potential of −310 mV (Hertle et al., 2013; Strand, Fisher, Davis, et al., 2016). It has also been proposed that *m*‐type TRXs, with redox potentials between −357 and −312 mV (Collin et al., 2003; Yoshida, Hara, & Hisabori, 2015), reduce an intermolecular disulfide in PGRL1 homodimers, and subsequently, the released monomeric PGRL1 may function as the ferredoxin‐plastoquinone reductase (Hertle et al., 2013). Here, we confirm the previously reported transient reduction of PGRL1 during dark‐to‐light transitions (Hertle et al., 2013), but NTRC overexpression does not intervene in the reduction (Supporting Information Figure S6A). Moreover, TRX‐*m*1 but not NTRC interacts with PGRL1 in BiFC (Figure 9). Our results thus support the hypothesis that TRX‐*m* is a primary reductant of PGRL1. Crosstalk between NTRC and FTR‐dependent systems (Nikkanen et al., 2016; Thormählen et al., 2015; Toivola et al., 2013), and the interaction of NTRC with TRX‐*m*1 in BiFC (Nikkanen et al., 2016), further support the interpretation that the activation of PGR‐dependent CEF is indirectly increased in NTRC‐overexpressing plants through enhancement of TRX‐*m* reduction. This would also be in line with the steady‐state *pmf* increase observed in OE‐NTRC *ndho* in comparison to WT (Figures 5a and 7a).

The NTRC overexpression may also affect the function of PGR5 in a way that is independent of its involvement in CEF. Redox regulation of PGR5 may occur to control its association with the ATP synthase during dark‐to‐light and low‐to‐high light transitions, and thereby allow inhibition of the ATP synthase in an unknown mechanism, as suggested earlier (Kanazawa et al., 2017; Tikkanen et al., 2015). Such a mechanism would result in acidification of the lumen and induction of NPQ, allowing dissipation of excess excitation energy from the electron transfer chain until CBC is activated. This hypothesis is supported by the impaired abilities of *pgr5* and OE‐NTRC *pgr5* to control the activity of the ATP synthase at early stages of dark‐light transitions and upon transitions to high light intensities (Figures 5d and 7d, Avenson, Cruz, Kanazawa, & Kramer, 2005). Furthermore, the elevated NTRC content in leaves caused decreased thylakoid proton conductivity upon increases in light intensity (Figure 7b,d), suggesting that NTRC controls the PGR5‐dependent downregulation of proton efflux from the lumen. This is supported by the identification of PGR5 as a potential NTRC interactor (Table 1, Figures 3 and 9).

The *pgr5* mutant is known to suffer from an impaired ability to induce ΔpH‐dependent photosynthetic control at Cyt *b*_*6*_ *f* upon shifts to high irradiance, possibly due to loss of the *pmf* (Suorsa et al., 2013; Takagi & Miyake, 2018). Interestingly, recovery of *pmf* in high light through NTRC overexpression (Figure 7) was not sufficient to prevent excess electron flow to PSI in *pgr5* background (Figure 8). This supports the hypothesis that the PGR5 protein is directly required to control linear electron flow (Suorsa et al., 2013; Takagi & Miyake, 2018; Tikkanen et al., 2015).

The initial strong *pmf* increase in OE‐NTRC after onset of growth light illumination was evident also in both the OE‐NTRC *ndho* and OE‐NTRC *pgr5* plants (Figure 5a,b) indicating that this *pmf* peak is not caused by CEF. More likely, the initial *pmf* results from dark‐activation of the CBC enzymes in plants overexpressing NTRC (Nikkanen et al., 2016), which provides an enhanced ability of the stroma to accept electrons from the PETC upon dark‐to‐light transition and consequently enhances proton pumping to the lumen.

### 3.5 Cooperative regulation of photosynthetic electron transfer and carbon fixation by chloroplast thioredoxin systems

**Figure 10:**
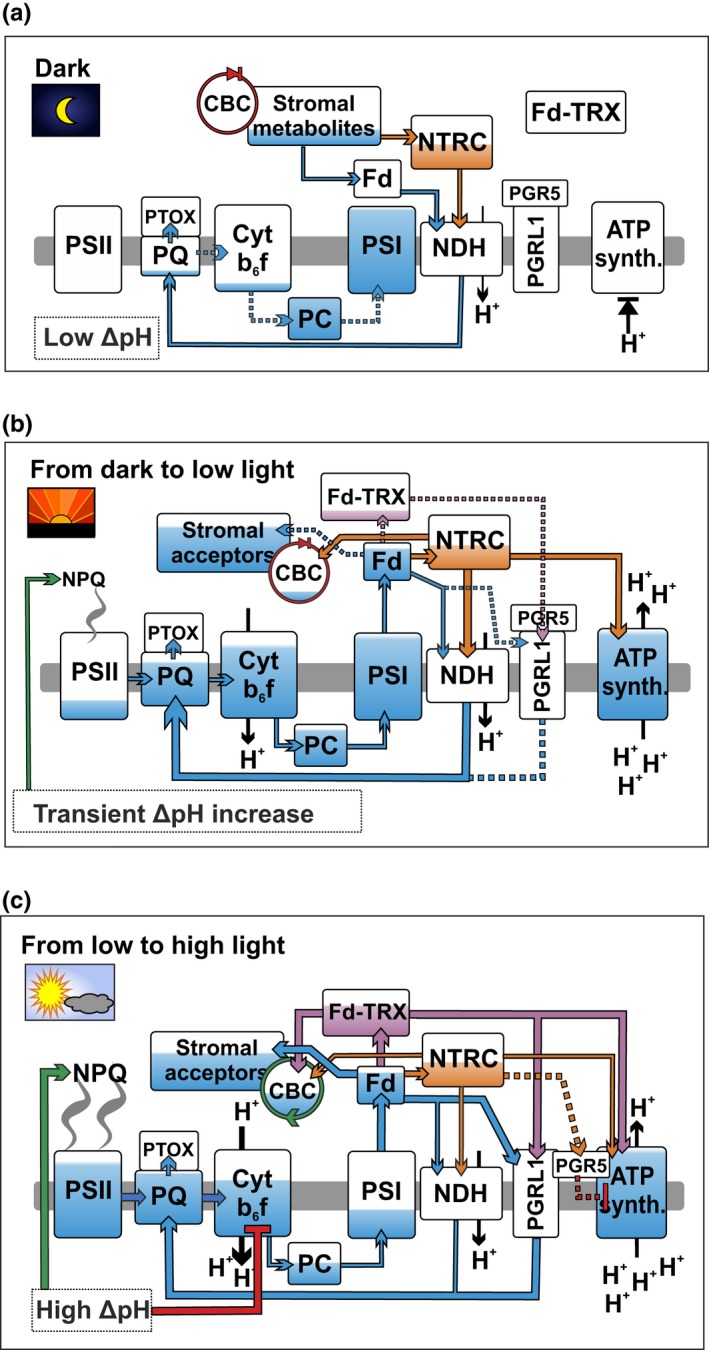
A schematic model of the role of chloroplast TRX systems in regulating CEF and the *pmf* during dark‐to‐light transitions and fluctuations in light intensity. (a–c) Dark‐adapted leaves (a), transition from dark to low light (b) and transition from low to high light (c). Blue color indicates the approximate reduction levels of different photosynthetic redox components based on data in the current paper (Figures 1, 2, 8, and 9) as well as other reports (Yoshida, Hara and Hisabori, 2015; Nikkanen et al., 2016; Schreiber, 2017). Green and red arrows indicate activating and inhibitory effects, respectively, while orange color represents the thiol regulation by NTRC and purple by the Fd‐TRX system. Thicker lines depict stronger effect than thin and dotted lines. For details see the text. The arrow to NPQ refers to the induction of the qE component of NPQ due to acidification of the lumen (Demmig‐Adams et al., 2012). The arrows representing reduction of PQ by the NDH complex and PGRL1 have been drawn through the lumen only to increase clarity of the illustration

Light‐dependent reductive activation of the ATP synthase, the CBC, and the NADP‐malate dehydrogenase (NADP‐MDH) by TRXs has been well established for several decades (reviewed in Buchanan, 2016). More recently, knowledge of TRX‐mediated control has been extended to various regulatory and photoprotective mechanisms of photosynthesis, including regulation of state transitions (Rintamäki, Martinsuo, Pursiheimo, & Aro, 2000; Shapiguzov et al., 2016), NPQ (Brooks, Sylak‐Glassman, Fleming, & Niyogi, 2013; Da et al., 2017; Hall et al., 2010; Naranjo et al., 2016) and CEF (Courteille et al., 2013; Hertle et al., 2013; Strand, Fisher, Davis, et al., 2016). We propose here a model, comprising a cooperative function of the two chloroplast TRX systems with distinct reductants and redox potentials that allows the maintenance of redox balance between the two photosystems and stromal metabolism during fluctuations in light conditions. This is achieved through dynamic regulation of the activities of the ATP synthase, NPQ, the NDH complex, PGRL1/PGR5 as well as the LHCII kinase STN7 by reversible thiol modifications. We propose a specific role for NTRC in regulating NDH‐CEF, the ATP synthase and CBC enzymes in low light, dark‐to‐light transitions, and during sudden increases in light intensity, as schematically depicted in Figure 10.

In darkness, a proportion of NDH complexes in the thylakoid membrane is activated by NTRC, and moderate chlororespiration from NDH to PTOX occurs. Due to an inactive ATP synthase and proton pumping activity of NDH, a weak proton gradient over the thylakoid membrane is established. Redox‐regulated CBC enzymes are inactive, causing PC and P700 to be reduced (Schreiber, 2017) due to lack of electron acceptors in the stroma. In OE‐NTRC, chlororespiration via NDH to the PQ pool is elevated due to an increased amount of active NDH complexes. This leads to increased protonation of the lumen and higher reduction of the PQ pool. Proportions of the ATP synthase and CBC enzyme pools are also activated due to high NTRC content.

Upon transition from dark to low light, the ATP synthase pool is fully reduced and the CBC enzyme pool partially reduced by NTRC in WT plants. Delay in activation of the CBC enzymes causes, however, a reduction of the PQ pool due to scarcity of stromal acceptors limiting electron transfer. NTRC contributes to the activation of NDH‐dependent CEF, which alleviates electron pressure at PSI and transiently increases *pmf* and induces NPQ. In OE‐NTRC, P700 and PC are effectively oxidized upon onset of low illumination, as the acceptor side limitation is negligible due to fully active NDH‐dependent CEF, ATP synthase, and redox‐activated CBC enzymes. This results in an elevated ΔpH and faster induction of NPQ in comparison to WT at the initial phase of illumination. At steady state, NPQ is lower than in WT despite high ΔpH, suggesting downregulation of NPQ by thioredoxin via a ΔpH‐independent mechanism, as reported previously by Brooks et al. (2013).

When a leaf is shifted from low to high irradiance, both TRX systems become fully active, and the CBC enzymes as well as the PGR‐dependent CEF are fully activated. NTRC affects PGR5‐dependent inhibition of the ATP synthase, which contributes to the accumulation of protons in the lumen. Consequently, NPQ and downregulation of electron transfer at Cyt *b6f* are induced. Electrons are effectively pulled from PSI, and the donor side is limiting electron transfer. In OE‐NTRC, increased reduction in PGR5 likely leads to stronger downregulation of the ATP synthase. This, together with proton pumping by constantly active NDH and possibly through increased TRX‐*m*‐mediated activation of the PGR‐dependent pathway, results in high *pmf*. NPQ is, however, lower than in WT due to ΔpH‐independent downregulation of NPQ by overexpressed NTRC.

## 4 MATERIALS AND METHODS

### 4.1 Plant material and growth conditions

Experiments have been carried out with *Arabidopsis thaliana* wild‐type (WT) lines of the Columbia ecotype (Col‐0 and Col‐*gl1*), and with the following transgenic lines: NTRC overexpression line (Toivola et al., 2013), T‐DNA knockout mutants of *NTRC* (At2g41680, SALK_096776) (Lepistö et al., 2009), *NDH‐O* (At1g74880, SALK_ 068922) (Rumeau et al., 2005) and *STN7* (At1g68830, SALK_073254) (Bellafiore et al., 2005) as well as the *pgr5* mutant (At2g05620) (Munekage et al., 2002). The plants were grown in a photoperiod of 8 hr light/16 hr darkness at 23°C under 200 μmol of photons m^−2^ s^−1^ for all experiments except for the measurements shown in Supporting Information Figure S2A, for which plants were grown in a 12 hr/12 hr photoperiod under 130 μmol m^−2^ s^−1^. Wild‐type tobacco **(** *Nicotiana benthamiana*) plants used in BiFC tests were grown under 130 μmol photons m^−2^ s^−1^ at 23°C in a 16 hr light/8 hr dark photoperiod. The OE‐NTRC *ndho* and OE‐NTRC *pgr5* lines were generated by *Agrobacterium tumefaciens* and floral dipping‐mediated transformation of the *ndho* knockout and *pgr5* mutant lines, respectively, with the NTRC overexpression construct as described previously (Toivola et al., 2013). The OE‐NTRC *ndho* and OE‐NTRC *pgr5* plants used in the experiments were heterozygous T2 generation plants that were selected on agar plates with 0.5X Murashige–Skoog medium (MS) (Murashige & Skoog, 1962) and 50 μg/ml kanamycin. The plants were subsequently transferred to soil and grown in an 8 hr light/16 hr darkness photoperiod at 23°C under 200 μmol of photons m^−2^ s^−1^ for 4 weeks before usage in the experiments. As control, OE‐NTRC plants were similarly selected on kanamycin‐containing plates while WT Col‐0 and WT Col‐*gl1* (ecotype of the *pgr5* mutant) plants were grown on 0.5X MS‐agar plates without antibiotics for an equivalent time.

### 4.2 Determination of H_2_O_2_ content in leaves

The hydrogen peroxide content in leaves was estimated by staining with diaminobenzidine (DAB), as previously described in Lepistö et al. (2013). Detached leaves from 4‐week‐old WT, *ntrc,* and OE‐NTRC plants were incubated overnight in darkness in 0.1 mg ml^−1^ solution of diaminobenzidine (DAB; Sigma‐Aldrich) (pH 3.8), after which the leaves were illuminated with either 40 or 200 μmol photons m^−2^ s^−1^ for 1 hr. Chlorophyll was then bleached by incubating the leaves in ethanol and subsequently photographed. Image J software (Schneider, Rasband, & Eliceiri, 2012) was used to quantify the intensity of the staining.

### 4.3 Measurement of chlorophyll a fluorescence and P700 and Fd redox changes

The postillumination chlorophyll a fluorescence rise (PIFR) was measured from detached leaves with the Multicolor‐PAM fluorometer (Walz). A 480‐nm measuring beam at an intensity of 0.2 μmol photons m^−2^ s^−1^ was used to measure fluorescence changes after illumination of dark‐adapted (30 min) leaves with 67 μmol photons m^−2^ s^−1^ of white actinic light for 500 s, with saturating pulses of 800 ms (10,000 μmol photons m^−2^ s^−1^) in the beginning and at 400 s to determine Fm and Fm’. For Figure 2c, saturating pulses were administered at 20‐s intervals for the first minute, 30‐s intervals for the second minute, and at 45‐s intervals for the rest of the experiment in order to more closely follow photosynthetic parameters such as NPQ and Y(II). The actinic light was then switched off and the changes in chlorophyll a fluorescence in the dark were observed for 300 s. A 10‐s pulse of far red light was then given to fully oxidize the PQ pool, and the subsequent rereduction of the PQ pool was detected through a rise in Chl fluorescence. All values were normalized to Fm.

The OJIP transients were recorded with the Multicolor‐PAM from dark‐adapted (30 min) leaves and from leaves preilluminated with far red light (intensity setting 15) for 6 s, according to the method described by (Toth et al., 2007). A saturating pulse of 3,000 μmol photons m^−2^ s^−1^ and measuring light at 440 nm were used in the measurements. The initial slopes of the transients in Supporting Information Table S1 were calculated from F/Fm values between 50 and 150 μs.

The Dual‐PAM‐100 (Walz) was used to simultaneously record the Chl a fluorescence and P700‐dependent difference in absorbance at 875 and 830 nm during transitions from dark to 166 μmol photons m^−2^ s^−1^ (Figure 6) and during a light regime where a 620 nm AL fluctuates between 39 and 825 μmol photons m^−2^ s^−1^ (Figure 8). Saturating pulses were administered at 10‐ or 15‐s intervals for the measurements in Figure 6 and at 15‐s intervals for the first minute after onset of illumination, and at 20‐s intervals thereafter for Figure 8. Because *ntrc* leaves are very small in size and low in chlorophyll content, it was in some cases necessary to record from two or three leaves simultaneously to obtain a P700 signal of sufficient quality. The parameters shown were calculated with the Dual‐PAM‐100 software as described by Bilger and Björkman (1990), Klughammer and Schreiber (2008a,b) and Kramer, Johnson, Kiirats, and Edwards (2004).

For determination of Fd redox state, the Dual/Klas‐NIR (Walz) spectrometer was used to record the four absorbance differences between 785 and 840, 810 and 870, 870 and 970, as well as 795 and 970 nm, from which the redox changes in P700, PC, and Fd were deconvoluted as described by Klughammer and Schreiber (2016) and Schreiber (2017). A similar illumination and postillumination regime was used as described above for the measurement of PIFR, with the exception that dark‐adapted leaves were illuminated with 61 μmol photons m^−2^ s^−1^ of 630 nm instead of white actinic light. Measured Fd redox changes were then normalized to the maximum level of Fd reduction, which was determined according to Schreiber and Klughammer (2016).

### 4.4 Measurement of electrochromic shift (ECS)

In order to measure the magnitude and kinetics of *pmf* formation, changes in the electrochromic shift (ECS, P515) signal were recorded with the Dual‐PAM‐100 and the P515/535 accessory module (Walz) (Klughammer, Siebke, & Schreiber, 2013; Schreiber & Klughammer, 2008). A dual beam difference signal between 550 and 515 nm was used to avoid distortion of results by scattering effects. A measuring light at a 2,000‐Hz pulse frequency was used in all ECS measurements. For the dark‐to‐light and low‐to‐high light transition measurements in Figures 5 and 7, plants were first dark‐adapted for 30 min. A single‐turnover saturating flash (20 μs) of 14,000 μmol photons m^−2^ s^−1^ was then applied to obtain ECS_ST_, a maximum absorbance change value that was used to normalize all results to account for differences in leaf thickness and chlorophyll content between individual leaves and lines (Kramer & Crofts, 1989). The obtained values of ECS_ST_ were in good correlation with the differences in chlorophyll content in OE‐NTRC and *ntrc* lines reported previously (Toivola et al., 2013). In order to distinguish the light‐induced ECS change (ECS_T_) from signal drift and baseline change, dark intervals of 250 ms were applied at the following time points after the onset of AL illumination: 0.8; 2.7; 4.7; 6.7; 8.7; 10.7; 12.7; 16.7; 20.7; 24.7; 28.7; 32.7; 36.7; 40.6; 44.7; 48.7; 52.7; 56.7; 60.7; 80.6; 100.5; 120.5; 140.5, and 160.5 s after onset of illumination. Additionally, during the shift from low to high irradiance (Figure 7), dark intervals were applied at 1.1; 5.1; 9.1; 13.1; 23.1; 33.1; 43.1; 53.1; 73.1; 93.1; 113.1; 133.1, and 153.1 s after the increase in light intensity. ECS_T_ was calculated as the difference between total ECS in light and an Y_0_ value obtained from the first‐order exponential fit to the decay kinetics of the ECS signal during a dark interval. Total *pmf* was then calculated as ECS_T_/ECS_ST_. The g_H_+ parameter, describing thylakoid membrane conductivity to protons, was calculated as the inverse of the time constant of a first‐order exponential fit to ECS decay kinetics during a dark interval (Avenson et al., 2005; Cruz, Sacksteder, Kanazawa, & Kramer, 2001; Cruz et al., 2005). The *v*_H+_ parameter indicating the rate of proton flux over the thylakoid membrane and corresponding to the initial slope of the decay of the ECS signal upon cessation of illumination was calculated as *pmf* x *g*_H+_ (Cruz et al., 2005). Partitioning of total *pmf* to its components ΔpH and ΔΨ was determined from the light‐off response of the ECS signal (Cruz et al., 2001) after 3 min of illumination, also using the Dual‐PAM ECS module as described by Schreiber and Klughammer (2008). Same settings were used for the determination of *pmf* partitioning as for the dark‐to‐light and low‐to‐high light transition measurements.

### 4.5 Protein extraction, alkylation of thiols, and SDS‐PAGE

Proteins and thylakoids were isolated as previously described (Lepistö et al., 2009), while chlorophyll content was determined according to (Porra, Thompson, & Kriedemann, 1989) and protein content with the Bio‐Rad Protein Assay kit. For determination of the redox states of TRX‐regulated proteins, leaf proteins were precipitated with trichloroacetic acid (TCA) and free thiols in proteins alkylated with N‐ethylmaleimide (NEM, Sigma‐Aldrich). After alkylation protein disulfides were reduced with dithiothreitol (DTT, Sigma‐Aldrich) and subsequently produced thiols were alkylated with methoxypolyethylene glycol maleimide M_n_ 5000 (MAL‐PEG, Sigma‐Aldrich) as described earlier (Nikkanen et al., 2016). Sodium dodecyl sulfate polyacrylamide gel electrophoresis (SDS‐PAGE) and immunoblotting was performed as reported in (Nikkanen et al., 2016). For running the MAL‐PEG samples precast 4%–20% Mini‐PROTEAN TGX gels (Bio‐Rad) were used, except for the gel in Figure 1a,b, and Supporting Information Figure S1A, where a 12% polyacrylamide gel was used. PVDF membranes were probed with antibodies raised against NTRC (Lepistö et al., 2009), D1 (Research Genetics, Inc (Thermo Fisher)), PsaB (Agrisera, AS10 695), Cyt *f* (kindly provided by L. Zhang), PTOX (kindly provided by M. Kuntz), NdhH (Agrisera), NdhS (Agrisera), CF_1_γ (Agrisera, AS08 312), PGRL1 (Agrisera, AS10 725), PGR5 (Agrisera) or phosphothreonine (P‐Thr) (New England Biolabs). Membranes were then treated with a horseradish peroxidase (HRP)‐conjugated goat anti‐rabbit secondary antibody (Agrisera, AS09 602) for 2 hr. All immunoblots shown are representative of at least three biological replicates. Quantifications of protein content shown in Figure 3b were performed using the ImageJ software (Schneider et al., 2012) and normalized according to the intensity of Li‐Cor Revert Total Protein Stain. Statistical significance was determined using two‐tailed Student's *t* tests for unequal variances with *p*‐values below 0.05 interpreted as statistically significant.

### 4.6 Coimmunoprecipitation and Mass spectrometry

For coimmunoprecipitation (Co‐IP), WT, *ntrc*, and OE‐NTRC leaves were frozen in liquid N_2,_ lysed in Pierce IP Lysis buffer containing 1% NP‐40 detergent (Thermo‐Fisher), and immunoprecipitated in a resin containing NTRC‐specific antibody using the Pierce Co‐IP kit (Thermo‐Fisher) with an affinity‐purified NTRC‐specific antibody, as described previously (Nikkanen et al., 2016). Co‐IP eluates were denatured and purified by SDS‐PAGE in a 6% acrylamide gel with 6 M urea, subjected to in‐gel tryptic digestion and the extracted peptides analyzed with the Q Exactive Hybrid Quadruple‐Orbitrap mass spectrometer (Thermo‐Fisher Scientific) in DDA mode as previously described (Trotta, Suorsa, Rantala, Lundin, & Aro, 2016). MS/MS spectra were analyzed with an in‐house installation of Mascot (v.2.4) (Matrix Science) search engine and analyzed with Proteome Discoverer (v.1.4) Software (Thermo Scientific), restricting the search to the nonredundant database TAIR10 supplemented with most common laboratory contaminants (Trotta et al., 2016). Peptides were validated by Decoy Database Search, with target false discovery rates (FDR) set to be below 0.01 (strict) or below 0.05 (relaxed).

### 4.7 BiFC tests

Bimolecular fluorescence complementation tests (BiFC) were performed as described in (Nikkanen et al., 2016). For the current study, coding sequences of PGR5, PGRL1a, and NdhS obtained from Arabidopsis Biological Resource Center (ABRC) were cloned into pSPYNE‐35S and pSPYCE‐35S binary vectors (Walter et al., 2004), and the resulting constructs were checked by sequencing. Primer sequences used for cloning are listed in Supporting Information Table S6. Imaging of YFP and chlorophyll autofluorescence from *N. benthamiana* leaves infiltrated with *Agrobacterium tumefaciens* strain GV3101 carrying the appropriate binary vectors was performed with a Zeiss LSM780 laser scanning confocal microscope 3 days after infiltration. The negative result between PGRL1:YFP‐N and NTRC:YFP‐C also serves as a negative control.

### 4.8 Multiple alignment of amino acid sequences

Amino acid sequences of NdhH, Ndh48, NdhS, NdhJ, and Ndh45 in *Arabidopsis thaliana* and, as available*,* in *Populus trichocarpa, Vitis vinifera, Glycine max, Solanum lycopersicum, Oryza sativa, Sorghum bicolor, Brachypodium distachion, Physcomitrella patens, Selaginella moellendorffii*, and *Synechocystis PCC 6803* were obtained from the UniProtKB database and aligned with the Clustal Omega 1.2.4 online alignment tool (Sievers et al., 2011) using default settings.

## AUTHOR CONTRIBUTIONS

L.N. and E.R. designed the research, L.N., J.T., and A.T. performed the research, L.N., J.T., A.T., M.T., and E.R. analyzed the data, L.N. and E.R. wrote the article with input from E.‐M.A., M.T., A.T., M.G.D., and J.T.

## ACCESSION NUMBERS

The Arabidopsis Genome Initiative locus identifiers (AGI) used in this paper are listed in Table 1, Supporting Information Table S2–S6 and Dataset S1.
