# Supplementary material for: Regulation of cyclic electron flow by chloroplast NADPH‐dependent thioredoxin system
Source: Plant Direct. 2018 Nov 7;2(11):e00093. doi: 10.1002/pld3.93 (PMC6508795; doi:10.1002/pld3.93)
Supplement: Supplementary file 2 [file PLD3-2-e00093-s002.pdf]

SUPPLEMENTAL MATERIAL

Regulation of cyclic electron flow by chloroplast NADPH-dependent thioredoxin system

Lauri Nikkanen, Jouni Toivola, Andrea Trotta, Manuel Guinea Diaz, Mikko Tikkanen, Eva-Mari Aro and Eevi Rintamäki*

Molecular Plant Biology, Department of Biochemistry, University of Turku, FI-20014 Turku, Finland

Short title: Redox-regulation of the thylakoid NDH complex

***Corresponding author:**
Eevi Rintamäki
evirin@utu.fi
Molecular Plant Biology
Department of Biochemistry
University of Turku
FI-20014 TURKU
Finland
+358504309491

A

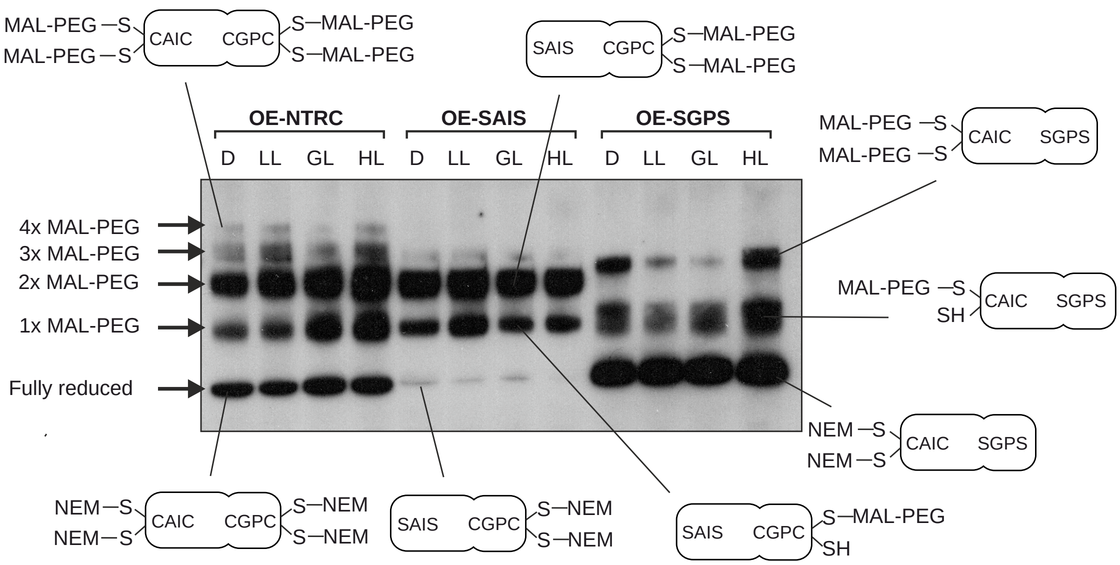

B

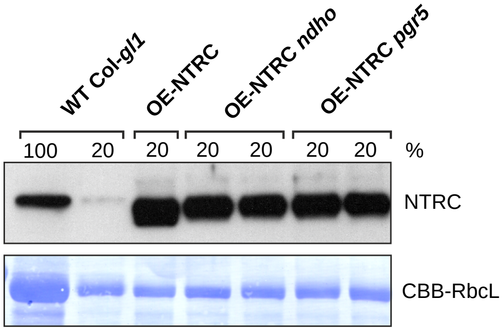

Supplemental Figure S1. Redox state and expression of NTRC in transgenic lines.

(A) Redox-state pattern of NTRC in leaves overexpressing NTRC. Total leaf proteins were extracted from leaves overexpressing NTRC (OE-NTRC) and from lines overexpressing mutated forms of NTRC where the redox-active cysteines in either the reductase domain (OE-SAIS) or in the TRX domain (OE-SGPS) have been mutated to serines (Toivola et al. 2013). In the mutated forms only 2 cysteine residues are available for alkylation by MAL-PEG molecules. The schematic drawings depict possible locations of MAL-PEG binding in specific bands. Leaves were incubated in darkness (D), or illuminated for 2 h in low light (LL, 40 µmol photons m^{-2} s^{-1}), growth light (GL, 200 µmol photons m^{-2} s^{-1}) or high light (HL, 800 µmol photons m^{-2} s^{-1}).

(B) Level of NTRC overexpression in OE-NTRC *ndho* and OE-NTRC *pgr5* plants detected by immunoblotting with an NTRC-specific antibody. 10 and 2 µg of soluble protein was loaded for WT and 2 µg for OE-NTRC, OE-NTRC *ndho* and OE-NTRC *pgr5*. Two individual plants were sampled for OE-NTRC *ndho* and OE-NTRC *pgr5*. Coomassie brilliant blue (CBB) staining was used as loading control.

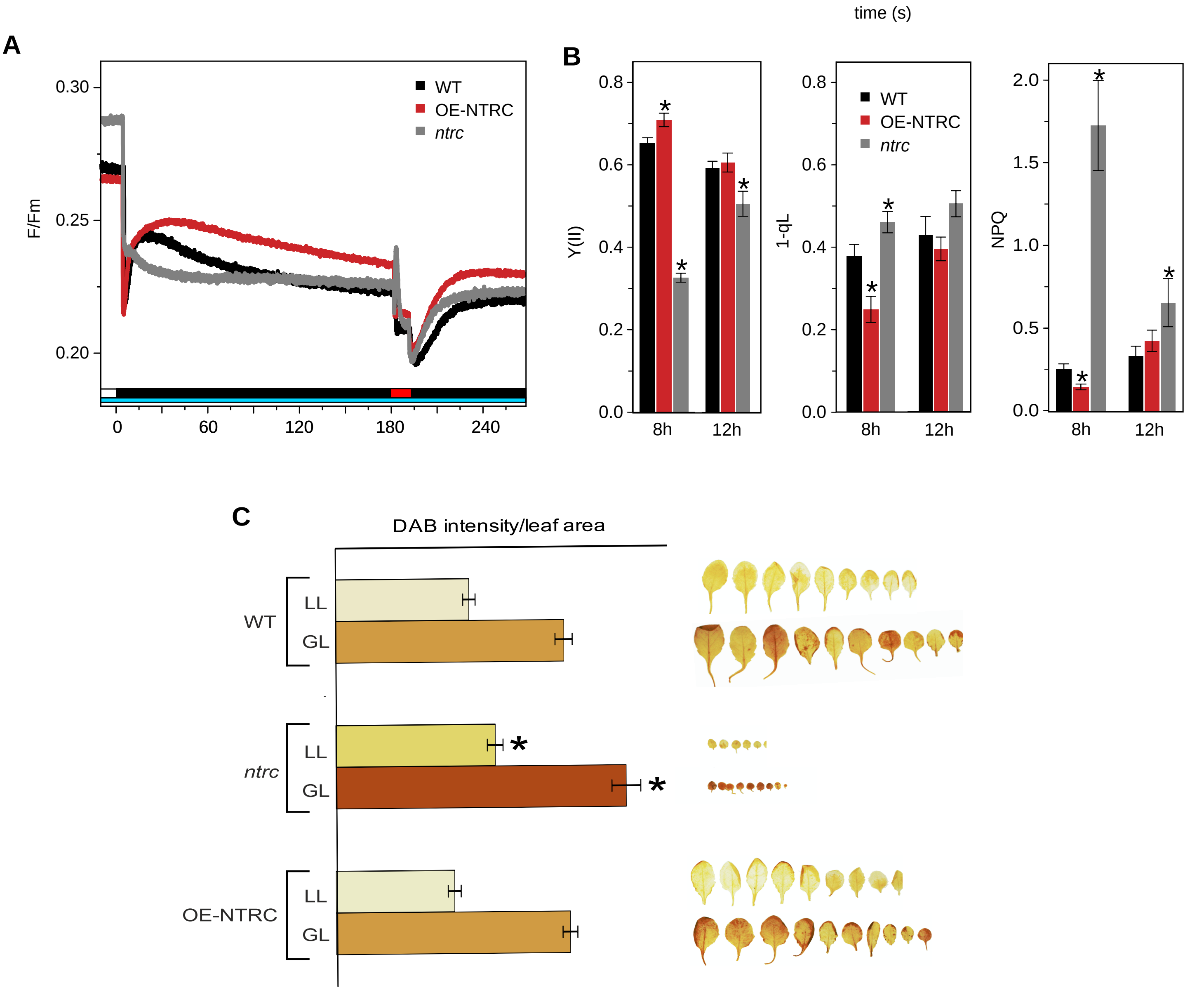

Supplemental Figure S2. Post-illumination fluorescence rise (PIFR) in plants grown in a 12h/12h photoperiod.

(A) PIFR in dark-adapted WT, OE-NTRC and *ntrc* plants grown in a 12h/12h photoperiod under 120 µmol photons m^{-2} s^{-1}. The cyan bar indicates exposure to a 480 nm measuring light of 0.28 µmol photons m^{-2} s^{-1}, the white bar depicts illumination with 67 µmol photons m^{-2} s^{-1} white light and the red bar shows the duration of a pulse of far red light. The curves are averages of measurements from 3–4 individual leaves.

(B) Quantum yield of PSII (Y(II)), redox state of the PQ pool (1-qL) and non-photochemical quenching (NPQ) at Fm' (after 7 min illumination) in plants grown either in an 8h or 12h photoperiod. The values are averages of 3–10 measurements ±SE, and statistically significant differences to WT according to Student's T-tests (P<0.05) are marked with *.

(C) Estimation of H_2O_2 content in WT, *ntrc* and OE-NTRC leaves after 1h illumination in low (LL) or growth light intensity (GL) based on the intensity of DAB staining. Values are represented as weighted (based on leaf area) averages of quantified DAB intensity from 12-19 individual leaves from 2 biological replicates ±SE, and statistically significant differences to WT according to Student's T-tests (P<0.05) are marked with *. Representative photographs of DAB-stained leaves are shown on the right.

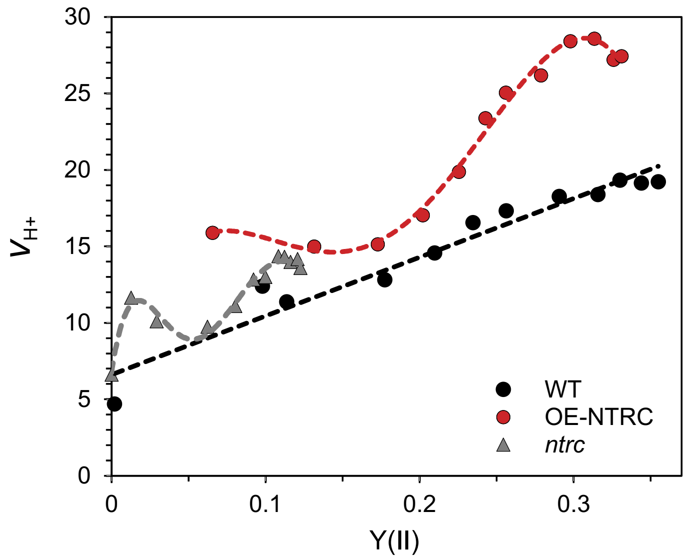

Supplemental Figure S3. Ratios between thylakoid proton flux and PSII quantum yield.

Proton flux (v_{H+}) over the thylakoid membrane during transitions from dark to light (166 µmol photons m^{-2} s^{-1}) as function of the quantum yield of PSII (Y(II)) under similar conditions. v_{H+} values were calculated from the data in Fig. 5 as *pmf* x g_{H+}. Values thus obtained were confirmed to correspond to the slope of the initial decay of the ECS signal upon cessation of illumination. Y(II) values were obtained from the data shown in Fig. 6. Linear (WT) or non-linear (OE-NTRC and *ntrc*) regression lines are shown.

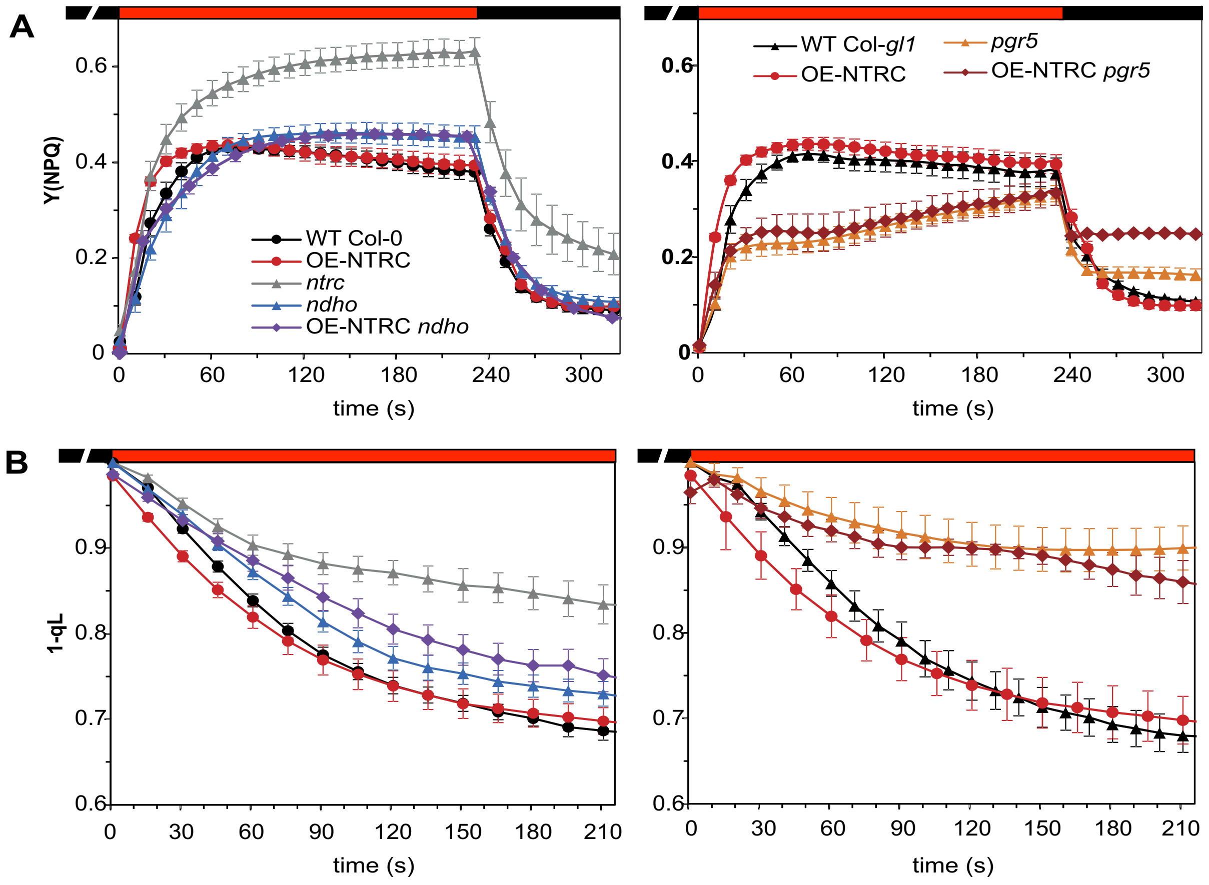

Supplemental Figure S4. NPQ and PQ pool redox state (1-qL) during dark-to-light transitions.

Induction of non-photochemical quenching (A) and redox state of the PQ pool (1-qL) (B) were calculated from Chl a fluorescence during transitions from dark to 166 µmol photons m^{-2} s^{-1} of actinic light in dark-adapted WT Col-0, *ntrc*, OE-NTRC, *ndho*, OE-NTRC *ndho*, WT Col-*gl1*, *pgr5* and OE-NTRC *pgr5* leaves. The graphs are averages of measurements from 4 to 9 individual leaves ±SE.

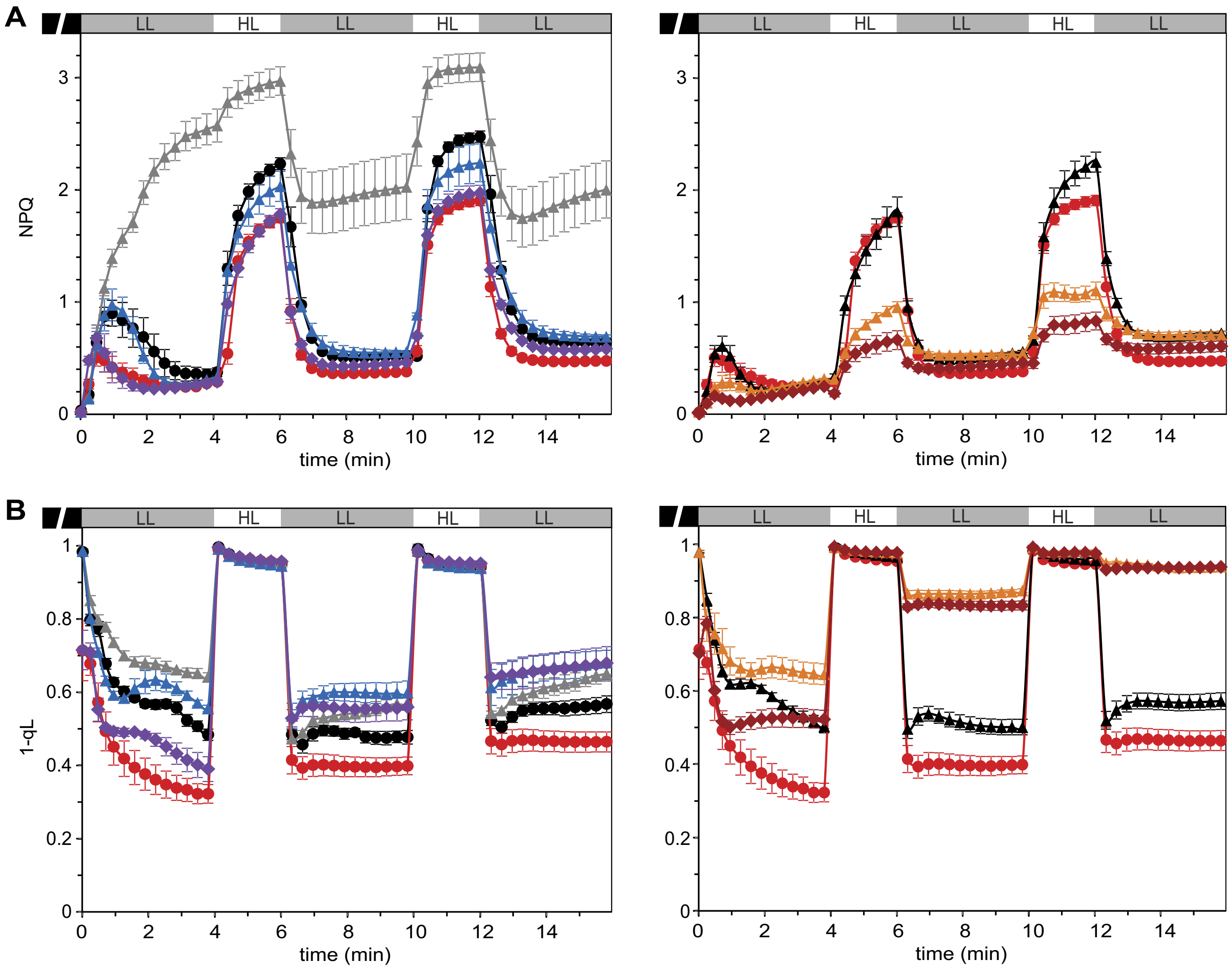

Supplemental Figure S5. NPQ and PQ pool redox state (1-qL) in fluctuating light.

Non-photochemical quenching (NPQ) (A) and redox state of the PQ pool (1-qL) (A) in light conditions fluctuating between periods of low actinic light (LL, 39 µmol photons $m^{-2} s^{-1}$) and high light (HL, 825 µmol photons $m^{-2} s^{-1}$) in WT Col-0, OE-NTRC, *ntrc*, *ndho*, OE-NTRC *ndho*, WT Col-*gl1*, *pgr5* and OE-NTRC *pgr5*. Five weeks old plants were dark-adapted for 30 min before measuring fluorescence from detached leaves. All values are averages of measurements from 3 to 10 individual leaves ±SE.

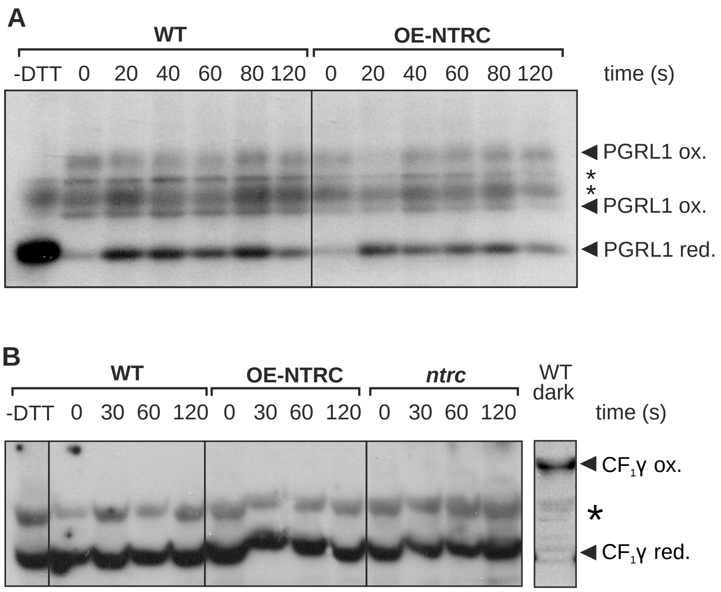

Supplemental Figure S6. *In vivo* redox states of PGRL1 and $CF_1\gamma$ during changes in light conditions.

(A) MAL-PEG assay to determine the *in vivo* redox state of PGRL1 during dark to light transitions. Samples were collected after illumination of dark-adapted leaves for 20, 40, 60, 80 and 120 seconds under growth light. –DTT is a control sample where free thiols were blocked with NEM but DTT was not added thereafter, preventing any alkylation by MAL-PEG.

(B) *In vivo* redox state of the ATP synthase γ subunit ($CF_1\gamma$) during transitions from low light (40 µmol photons m^{-2} s^{-1}) to high light (600 µmol photons m^{-2} s^{-1}) in WT, OE-NTRC and *ntrc* leaves. Prior to the light intensity shift plants grown under 200 µmol photons l m^{-2} s^{-1} were kept 30 min under low irradiance. Samples were taken after illumination for 0, 30, 60 and 120 seconds in high light. A dark-incubated control (WT dark) is seen in the right panel.* marks unspecific bands.

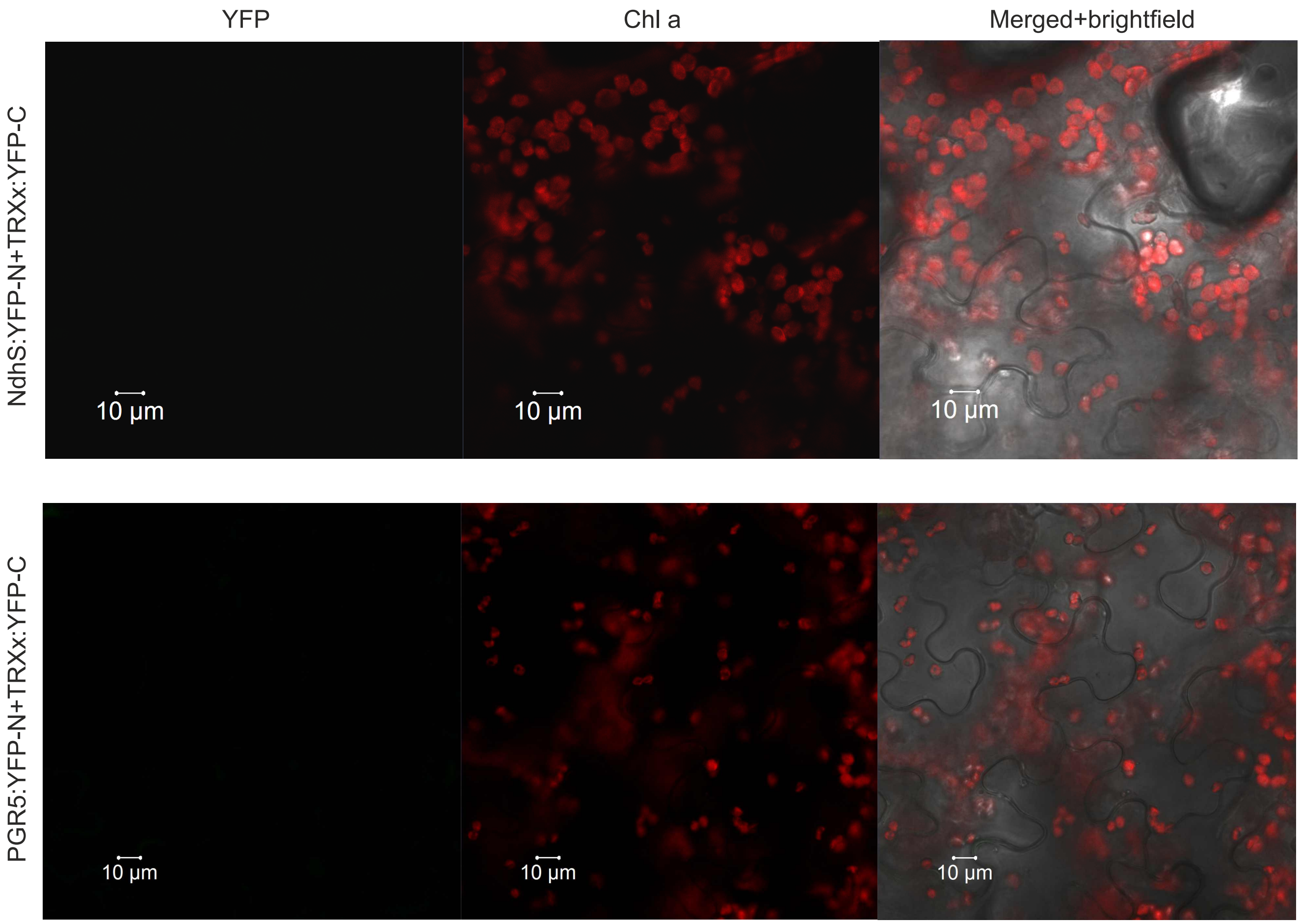

Supplemental Figure S7. Bimolecular fluorescence complementation (BiFC) tests between TRX-*x* and NdhS and PGR5.

The left panel shows yellow fluorescent protein (YFP) fluorescence in green, the middle panel Chlorophyll a autofluorescence in red and the right panel a merged image of YFP, chlorophyll and brightfield images. YFP-N and YFP-C indicate expression of fusion proteins including the N-terminal and C-terminal parts of YFP, respectively, in tobacco (*Nicotiana benthamiana*) leaves.

Supplemental Table S1. Parameters determined from OJIP transients of chlorophyll a fluorescence.

Apparent F_O values (F/Fm after 20 µs of light), integrated area above O-J phase (0-3 ms) ($A_{O\text{-}J}$) and the initial slopes of the O-J transients ($m_{50\text{-}150\ \mu s}$) in dark-adapted leaves of WT, OE-NTRC, *ntrc*, *pgr5* and *ndho* were calculated from the averaged curves in Figure 3.

**line**	**F_0 ($F_{20\mu s}$)**	**$A_{O\text{-}J}$**	**$m_{50\text{-}150\ \mu s}$**
WT	0.18	1.62	0.58
OE-NTRC	0.21	1.46	0.74
ntrc	0.26	1.29	1.20
pgr5	0.21	1.84	0.47
ndho	0.17	1.76	0.57

Supplemental Table S2. Screening of putative NTRC target proteins by Co-IP/MS.

One hundred chloroplast proteins present in WT and/or OE-NTRC eluates but absent from *ntrc* eluates are listed in this table in the order of their abundance in Co-IP eluates (total number of peptide spectrum matches in WT + OE-NTRC samples) (Supplemental Dataset 1). At least two unique peptides were detected of each protein. Previously published TRX targets are highlighted with blue (articles 3, 5, 6, 7, 8, and 9 in Suppl. references) and established NTRC interactors with red colour (articles 1, 2, 4, 10, and 11 in Suppl. references), while proteins involved in CEF are in bold. Functions of the proteins are provided based on the annotation in the TAIR database.

#	AGI code	Annotation	*ntrc*	WT	OE-NTRC	Function
1	**ATCG01110.1**	**NAD(P)H dehydrogenase subunit H**	**-**	**+**	**+**	**Cyclic electron flow**
2	**AT1G15980.1**	**NDH-dependent cyclic electron flow 1 (Ndh48)**	**-**	**+**	**+**	**Cyclic electron flow**
3	AT3G11630.1	2-Cysteine peroxiredoxin A/B[1]	-	+	+	Redox regulation
4	AT4G25080.4	magnesium-protoporphyrin IX methyltransferase[2]	-	+	+	Chl biosynthesis
5	AT5G43780.1	sulfate adenylyltransferase, ATP sulfurylase (APS4, ATPS4)	-	+	+	Sulfate assimilation, AA synthesis
6	AT3G27925.1	DegP protease 1[3]	-	+	+	proteolysis
7	AT2G42220.1	Rhodanese/Cell cycle control phosphatase superfamily protein	-	+	+	Cell cycle
8	AT2G40100.1	LHCB4.3, LIGHT HARVESTING COMPLEX PHOTOSYSTEM II	-	+	+	Photosynthesis
9	ATCG01120.1	chloroplast ribosomal protein S15	-	+	+	Translation
10	AT4G17090.1	chloroplast beta-amylase[4]	-	+	+	Starch degradation
11	AT1G69830.1	alpha-amylase-like 3[5]	-	+	+	Starch degradation
12	AT5G53580.1	ATPLR1, PLR1, PYRIDOXAL REDUCTASE 1	-	+	+	Carbon metabolism
13	AT5G03880.1	Thioredoxin family protein	-	+	+	Redox regulation
14	AT3G55610.1	delta 1-pyrroline-5-carboxylate synthase 2	-	+	+	AA biosynthesis
15	AT1G70820.1	phosphoglucomutase, putative	-	+	+	Carbon metabolism
16	AT4G22710.1	cytochrome P450, family 706, subfamily A, polypeptide 2	-	+	+	Secondary metabolism
17	AT3G54110.1	plant uncoupling mitochondrial protein 1	-	+	+	Photorespiration
18	AT4G05180.1	photosystem II subunit Q-2	-	+	+	Photosynthesis
19	AT2G04842.1	threonyl-tRNA synthetase, putative / threonine--tRNA ligase, putative	-	+	+	Translation
20	AT5G37510.1	NADH-ubiquinone dehydrogenase, mitochondrial, putative	-	+	+	Mt respiration
21	AT5G65220.1	Ribosomal L29 family protein	-	+	+	Translation
22	AT4G29670.1	ACHT2, ATYPICAL CYS HIS RICH THIOREDOXIN 2	-	+	+	Redox regulation
23	AT4G23650.1	CALCIUM-DEPENDENT PROTEIN KINASE 6, CDPK6, CPK3	-	+	+	Photosynthesis
24	AT1G58290.1	Glutamyl-tRNA reductase family protein; GLUTR, HEMA1[2]	-	+	+	Chl biosynthesis
25	AT1G58080.1	ATP phosphoribosyl transferase 1	-	+	+	AA biosynthesis
26	AT3G24430.1	HCF101	-	+	+	Photosynthesis
27	AT2G32730.1	26S proteasome regulatory complex, Rpn2/Psmd1 subunit	-	+	+	Proteolysis
28	AT5G11880.1	Pyridoxal-dependent decarboxylase family protein	-	+	+	aa synthesis
29	AT1G63970.2	2C-METHYL-D-ERYTHRITOL 2,4-CYCLODIPHOSPHATE SYNTHASE, MECPS	-	+	+	Carbon & secondary metabolism
30	AT5G40950.1	ribosomal protein large subunit 27	-	+	+	Translation
31	AT5G03290.1	isocitrate dehydrogenase V, IDH-V	-	+	+	Carbon metabolism
32	AT5G65840.1	Thioredoxin superfamily protein	-	+	+	Redox regulation
33	**AT4G23890.1**	**CHLORORESPIRATORY REDUCTION 31, CRR31, NdhS**	**-**	**+**	**+**	**Cyclic electron flow**
34	ATCG00330.1	chloroplast ribosomal protein S14	-	+	+	Translation
35	AT1G34000.1	OHP2, ONE-HELIX PROTEIN 2	-	+	+	Photosynthesis
36	AT1G18500.1	IPMS1, ISOPROPYLMALATE SYNTHASE 1, MAML-4,	-	+	+	AA synthesis
37	AT2G28800.4	ALB3, ALBINO 3	-	+	+	Cplast biogenesis & senescence
38	AT5G08540.1	unknown protein	-	+	+	Unknown
39	AT1G08520.1	CHLD, ALB-1V, ALB1, ALBINA 1, PDE166	-	-	+	Chl biosynthesis
40	AT1G06680.2	photosystem II subunit P-1	-	+	+	Photosynthesis
41	AT5G26830.1	Threonyl-tRNA synthetase	-	+	+	Translation
42	AT4G15110.1	cytochrome P450, family 97, subfamily B, polypeptide 3	-	+	-	Carbon metabolism
43	AT5G66420.1	unknown protein	-	+	-	Unknown
44	AT1G33780.1	unknown protein	-	+	+	Unknown
45	AT1G45201.2	triacylglycerol lipase-like 1	-	+	+	Lipid metabolism
46	AT1G48350.1	Ribosomal L18p/L5e family protein	-	+	+	Translation
47	AT1G09620.1	leucine-tRNA ligase	-	+	+	Translation

48	AT1G64190.1	6-PHOSPHOGLUCONATE DEHYDROGENASE 1, PGD1[6]	-	+	-	Carbon metabol.
49	AT5G14320.1	Ribosomal protein S13/S18 family	-	+	-	Translation
50	AT5G19940.2	Plastid-lipid associated protein PAP / fibrillin family protein	-	+	+	Lipid metabolism
51	AT1G50450.1	Saccharopine dehydrogenase	-	+	+	
52	AT3G20680.1	unknown protein	-	+	+	unknown
53	AT1G77060.1	Phosphoenolpyruvate carboxylase family protein	-	+	+	Carbon metabolism
54	AT5G27380.1	glutathione synthetase 2, GSH2	-	+	+	Redox regulation
55	AT1G51110.1	Plastid-lipid associated protein PAP / fibrillin family protein	-	+	+	AA biosynthesis
56	AT3G10940.1	dual specificity protein phosphatase (DsPTP1) family protein	-	+	+	Starch degradation
57	AT5G47890.1	NADH-ubiquinone oxidoreductase B8 subunit, putative	-	+	+	Mitochondrial respiration
58	AT1G10760.1	GWD, GWD1, SEX1, SOP, SOP1, STARCH EXCESS 1[7]	-	+	-	Strach degradation
59	AT4G26900.1	HIS HF, HISN4	-	+	+	AA synthesis
60	AT4G21280.1	photosystem II subunit QA	-	-	+	Photosynthesis
61	ATCG00820.1	ribosomal protein S19	-	+	+	Translation
62	AT2G34460.1	NAD(P)-binding Rossmann-fold superfamily protein	-	+	+	unknown
63	AT1G56500.1	SOQ1, SUPPRESSOR OF QUENCHING 1	-	+	+	Redox regulation
64	AT5G54600.1	PLASTID RIBOSOMAL PROTEIN L24, RPL24	-	+	+	translation
65	AT2G21170.1	triosephosphate isomerase, TPI[6]	-	+	-	Carbon metabolism
66	AT4G35630.1	phosphoserine aminotransferase	-	+	-	AA synthesis
67	AT4G31780.2	monogalactosyl diacylglycerol synthase 1, MGD1, MGDA[8]	-	+	-	Lipid metabolism
68	AT3G04870.1	zeta-carotene desaturase, ZDS	-	+	+	Carbon metabolism, secondary metabilsm
69	AT5G14060.1	lysine-sensitive aspartate kinase CARAB-AK-LYS	-	-	+	AA synthesis
70	AT1G80480.1	plastid transcriptionally active 17	-	-	+	unknown
71	AT1G75460.1	ATP-dependent protease La (LON) domain protein	-	-	+	Proteolysis
72	AT1G18060.1	unknown protein	-	+	+	Unknown
73	AT1G50200.2	Alanyl-tRNA synthetase	-	+	+	Translation
74	AT2G30790.1	photosystem II subunit P-2	-	+	+	Photosynthesis
75	AT3G54210.1	Ribosomal protein L17 family protein	-	+	-	Tranlation
76	**AT3G01440.1**	**PsbQ-like 2**	**-**	**+**	**-**	**Cyclic electron flow**
77	AT1G02560.1	nuclear encoded CLP protease 5	-	+	+	Proteolysis
78	AT2G15620.1	nitrite reductase 1, NIR1[9]	-	+	+	Nitrogen metabolism
79	AT5G18660.1	PALE-GREEN AND CHLOROPHYLL B REDUCED 2, PCB2	-	+	+	Chl biosynthesis
80	AT3G59780.1	Rhodanese/Cell cycle control phosphatase superfamily protein	-	+	+	Cell cycle
81	**AT1G74880.1**	**NAD(P)H:plastoquinone dehydrogenase complex subunit O**	**-**	**+**	**+**	**Cyclic electron flow**
82	AT5G36170.2	high chlorophyll fluorescent 109, HCF109	-	+	+	
83	AT1G16880.1	ACR11, ACT DOMAIN REPEATS 11	-	+	+	AA metabolism
84	AT5G24300.1	ATSS1, SS1, STARCH SYNTHASE 1[10]	-	+	-	Starch synthesis
85	AT1G78140.1	S-adenosyl-L-methionine-dependent methyltransferases superfamily	-	+	-	
86	AT4G25450.3	non-intrinsic ABC protein 8	-	+	-	unknown
87	**AT5G21430.2**	**NADH dehydrogenase-like complex U, NdhU**	**-**	**+**	**-**	**Cyclic electron flow**
88	AT1G16410.2	BUS1, BUSHY 1, CYP79F1, CYTOCHROME P450 79F1, SPS1	-	+	-	Secondary metabolism
89	**AT2G05620.1**	**PROTON GRADIENT REGULATION 5, PGR5**	**-**	**+**	**-**	**Cyclic electron flow**
90	AT3G06050.1	peroxiredoxin IIF	-	+	+	Redox regulation
91	AT2G32920.1	PDI-like 2-3 , Protein disulfide-isomerase 2-3	-	+	+	Redox regulation
92	AT4G12830.1	alpha/beta-Hydrolases superfamily protein	-	+	+	unknown
93	AT3G55250.1	Putative calcium homeostasis regulator	-	-	+	Signalling
94	AT3G53900.1	uracil phosphoribosyltransferase	-	-	+	
95	AT1G77490.1	thylakoidal ascorbate peroxidase	-	+	+	Redox regulation
96	AT5G11450.1	PsbP domain-containing protein 5, PPD5	-	-	+	
97	AT4G34120.1	Cystathionine beta-synthase (CBS) family protein, CBSX2[11]	-	-	+	Redox regulation
98	AT1G31800.1	LUTEIN DEFICIENT 5	-	+	+	Carotenoid biosynthesis
99	AT3G02780.1	isopentenyl pyrophosphate:dimethylallyl pyrophosphate isomerase 2	-	+	+	Chl biosynthesis
100	AT4G37200.1	Thioredoxin superfamily protein HCF164	-	+	+	Redox regulation

Supplemental Table S3. Multiple alignment of NdhS amino acid sequences in embryophytes.

Sequences from *Physcomitrella patens* (PHYPADRAFT_188716), *Glycine max* (GLYMA03G01720), *Brachypodium distachion* (BRADI3G21280), *Sorghum bicolor* (Sb07g028880), *Oryza sativa* (OS07G0196200), *Solanum lycopersicum* (Solyc08g082400.1), *Arabidopsis thaliana* (AT4G23890) and *Vitis vinifera* (VIT_02s0025g01470). Conserved cysteine residues are in bold and highlighted with red rectangles. ***** signifies full conservation of a residue in the current dataset, while "**:**" and "**.**" signify conservation of residues with strongly and weakly similar properties, respectively.

```
Physcomitrella      MA--AMTGICRAPLSLALQRESSFWGSNVGVHQQQKSASSNSGGSTGGVRVGIRAEGFDF
Glycine             -----MSSFVALQGLHGSLLSSQFLGQDTLTHFHPRNKASSTIQ-NKPTTAQQPSAKFDM
Brachypodium        --------------MAPAPTTPSFLRPPPLPHHR---------V-R--LPPPPPSASFRL
Sorghum             --------------MAPPPTSSSFLRPPPLPHHP---------H-PRLH-FRPPSASFRV
Oryza               ---------------MAPTTPASFLRPPPLPHHH---------H-PRIVRLPPPSATFRV
Solanum             MASASSFQLSSLQIQTPPLKKSNFLGQSVNLNLS-SSVHTKSAM-KSSSNSVTPIAKFNL
Arabidopsis         ---MATSSITIPTIRTP-IHRSKFLGQTHQFSTVNRSVFPPPKQ-QSKLYQVKAMGKFNL
Vitis               ----MAYSFTVPSLQRPLPHKSHFLGQGHFPNNIQKASLSR----TRTPLPVKASAKFDL
                                           *                                 * .

Physcomitrella      WQVLGGRGLKGGEDGLKQEKTARVLQEAKKNLVVE-KKKKGSVEGNVEAAEGLPGTFNKE
Glycine             LQIVGGRGLCNGEAGLKQELKKQVGVDEKGQTSAT-SGKEQEEEEESTSVVATEDGFEKE
Brachypodium        AEILGGRGLCNGEVGIRKELSSTPTTPSTPTADSS-P---GGAAAEADPPAVDPDAFEKE
Sorghum             SEILGGRGLCNGEVGVRKELTSGSSAST---TTSS-PAPSPSPSTESPPPAVDLDAFDKE
Oryza               ADLLGGRGLCNGEVGIRKELASDSPAAPPSTTTSS-DEPAESPPPPPAASGVDPDAFDKE
Solanum             YEILGGRGLCNGEEGIEKELKKSISEEQKAVGSAAAAASDDDNQENKETGEIPEDGFEKE
Arabidopsis         WEVMGGRGLCNGEKGIEKELQRNIEDEQETSKAEN-NETERESDDSNLSFKVPEDGFEKE
Vitis               FGIMGGRGLCNGEEGLQQELKRNIEPAPSPDSVKD-E----EKPALAAVDDVPEDGFDKE
                      ::***** .** *:.:*                                   . *:**

Physcomitrella      LGGWTGGFPGGEKGLRQFVQSNPPPAKASQMSNEIRKLQDSISRPLKPRAPSPPLLMPGM
Glycine             LMGLTGGFPGGEEVHSG---------KPSSSKTKSSKN-LKLALSKKPKPPELPLLLPGM
Brachypodium        MMGLTGGFPGGEVGLKDFVAKNPPPPPKRTQPDGIAGS-AAVVPAERPRRPELPLFLPGM
Sorghum             MMGLTGGFPGGEVGLKDFVAKNPPPPRSKKSNSQLVAP-QATTLSAPPRTPELPLFLPGM
Oryza               MMGLTGGFPGGEVGLKDFVAKNPPPPKPAHRKGLA-----AAATVERPRAPELPLFLPGM
Solanum             MMGFTGGFPGGEKGLMKFIEKNPPPPPPKKTESSMVSG-FNQSLVKKPKPPELPLLLPGM
Arabidopsis         MMGLTGGFPGGEKGLKTFIEKNPPPPPPPPPAKQGSDA-SAVATDKKPKAPKLPLLMPGM
Vitis               LLGLTGGFPGGEKGLKQFLEKNPPPEKT------SGNI-IENARLRKPKPPELPLLMPGM
                    : * ********                                   *: *. **::***

Physcomitrella      TVKVISPSNPYFEFIGIVQRVTDGKVGVIFEGGNWDKLVSFKLQDLERTSQGPPMSNPKS
Glycine             IAIVKNPNNPFYMYCKIVQRITDG---------------------------PRMKNPKS
Brachypodium        IVLVKNPRNAYHMYCGIVQRVTDGKVGVLFEGGNWDRLITFGVDELEGREKGPPMVNPKS
Sorghum             VVLVKNPNNAYYMYCGIVQRVTDGKVAVLFEGGIWDRLITFNLDELEGREKGPPMVNPKS
Oryza               VVLVKNPNNAYHMYCGIVQRVTDGKVGVLFEGGIWDRLITFDLDELEGREKGPPMVNPKS
Solanum             IAIVKNSNNPYYMYCGIVQRITDGKAAVLFEGGNWDRLISFRLEELERREKGPPMVNLKS
Arabidopsis         IAIVKNQNSPYHMYCGIVQRITDGKAGVLFEGGNWDRLITFRLEELERREKGPPGKNPKS
Vitis               IAIVKNPNNPFYMYCGIVQRITDGKAGVLFEGGNWDRLITFRLEELQRRDKGPPMKNPKS
                     . * .  . :. :  ****:***                            *   * **

Physcomitrella      AILERMIVPEGTSSPEGASS-----------------
Glycine             AVLKPFLEKKS--------------------------
Brachypodium        VVLEALVADLADDTEAEE----TEKKEEEAGAAAAKA
Sorghum             VVLEDIVAQLEDDDDDKE-DEAAKKEKEPEGAAAAA-
Oryza               VLLESLAAEMEDDVAKEEEGEEAKKKKEEEGTAAAA-
Solanum             VILEKMVEKSSEA------------------------
Arabidopsis         CILEPLIEQMQKEEAAP--------------------
Vitis               AILETLLEQEA--------------------------
```

Supplemental Table S4. Multiple alignment of NdhH amino acid sequences in photosynthetic organisms. Sequences from *Synechocystis PCC 6803* (slr0261), *Physcomitrella patens* (NDHH_PHYPA), Glycine max (NDHH_SOYBN), *Brachypodium distachion* (NDHH_BRADI), *Sorghum bicolor* (NDHH_SORBI), *Oryza sativa* (Osp1g00970), *Solanum lycopersicum* (NDHH_SOLLC), *Arabidopsis thaliana* (ATCG01110) and *Vitis vinifera* (NDHH_VITVI) were obtained aligned as described in the legend for Supplemental table 2.

```
Synechocystis       MTKIETRTEPMVLNMGPHHPSMHGVLRLIVTLDGEDVVDCEPVIGYLHRGMEKIAESRTN
Physcomitrella      --MLATKTKPMIVSMGPHHPSMHGVLRLIVTLDGENVIDCEPILGYLHRGMEKIAENRTI
Sorghum             MSLSLKRKDLMIVNMGPQHPSMHGVLRLIVTLDGEDVIDCEPILGYLHRGMEKIAENRSI
Brachypodium        MSLPLTRKDLMIVNMGPQHPSMHGVLRLIVTLDGEDVIDCEPILGYLHRGMEKIAENRTI
Oryza               MSLPLTRKDLMIVNMGPQHPSMHGVLRLIVTLDGEDVIDCEPILGYLHRGMEKIAENRTI
Glycine             MNISTTRKDFMIVNMGPHHPSMHGVLRLIVTLDGEDVIDCEPILGYLHRGMEKIAENRTI
Arabidopsis         MKRPVTGKDLMIVNMGPHHPSMHGVLRLIVTLDGEDVVDCEPILGYLHRGMEKIAENRAI
Solanum             MTAPTTRKDLMIVNMGPQHPSMHGVLRLIVTLDGEDVVDCEPILGYLHRGMEKIAENRTI
Vitis               MNVPATREDLMIVNMGPHHPSMHGVLRLIVTLDGEDVIDCEPILGYLHRGMEKIAENRTI
                         .  . *::.***:*****************:*:****::************.*:

Synechocystis       IMYVPYVSRWDYAAGMFNEAITVNAPEKLADIEVPKRAQYIRVIMLELNRIANHLLWLGP
Physcomitrella      VQYLPYVTRWDYLATMFTEAITVNAPEKLTNIQVPKRASYIRMIMLELSRVASHLLWLGP
Sorghum             IQYLPYVTRWDYLATMFTEAITVNAPEFLENIQIPQRASYIRVIMLELSRIASHLLWLGP
Brachypodium        IQYLPYVTRWDYLATMFTEAITVNAPEFLENIQIPQRASYIRVIMLELSRIASHLLWLGP
Oryza               IQYLPYVTRWDYLATMFTEAITVNAPEFLENIQIPQRASYIRVIMLELSRIASHLLWLGP
Glycine             IQYLPYVTRWDYLATMFTEAITVNGPEQLGNIQVPKRASYIRVIMLELSRIASHLLWLGP
Arabidopsis         IQYLPYVTRWDYLATMFTEAITVNGPEQLGNIQVPKRASYIRVIMLELSRIASHLLWLGP
Solanum             IQYLPYVTRWDYLATMFTEAITINGPEQLGNIQVPKRASYIRVIMLELSRIASHLLWLGP
Vitis               IQYLPYVTRWDYLATMFTEAITVNAPEQLGNIQVPKRASYIRVIMLELSRIASHLLWLGP
                    : *:***:**** * **.****:*.** * :*::*:**.***:*****.*:*.*******

Synechocystis       FMADVGAQTPFFYIFREREMIYDLWEAASGMRLINNNYFRVGGVAVDLPYGWNDKCEDFC
Physcomitrella      FMADIGAQTPFFYILREREMIYDLFEAATGMRMMH-NYFRIGGVAVDLPYGWIDKCLDFC
Sorghum             FMADLGAQTPFFYIFRERELIYDLFEAATGMRMMH-NYFRIGGVAADLPYGWMDKCLDFC
Brachypodium        FMADLGAQTPFFYIFRERELIYDLFEAATGMRMMH-NYFRIGGVAADLPYGWIEKCLDFC
Oryza               FMADLGAQTPFFYIFRERELIYDLFEAATGMRMMH-NYFRIGGVAADLPYGWIDKCLDFC
Glycine             FMADIGAQTPFFYIFRERELIYDLFEAATGMRMMH-NFFRIGGVATDLPYGWVDKCYDFC
Arabidopsis         FMADIGAQTPFFYIFREREFVYDLFEAATGMRMMH-NFFRIGGIAADLPYGWIDKCLDFC
Solanum             FMADIGAQTPFFYIFRERELIYDLFEAATGMRMMH-NYFRIGGVAADLPYGWIDKCLDFC
Vitis               FMADIGAQTPFFYIFRERELVYDLFEAATGMRMMH-NYFRIGGVAADLPHGWIDKCLDFC
                    ****:*********:****::***:***:***::: *:**:**:*.***:** :** ***

Synechocystis       DYFLPKVDEYEKLITNNPIFRRRVEGVGTVTREEAINWGLSGPMLRGSGVKWDLRKVDHY
Physcomitrella      DYFLPKVNEYERLITNNPIFLKRVEGIGIIGKEEAINWGLSGPMLRASGVQWDLRKVDHY
Sorghum             DYFLQGVVEYQQLITRNPIFLERVEGVGFISGEEAVNWGLSGPMLRASGIQWDLRKIDPY
Brachypodium        DYFLRGVVEYQQLITQNPIFLERVERVGFISGEEAVNWGLSGPMLRASGIRWDLRKVDLY
Oryza               DYFLRGVIEYQQLITQNPIFLERVEGVGFISGEEAVNWGLSGPMLRASGIQWDLRKVDLY
Glycine             DYFLTRIVEYQKLITRNPIFLERVEGVGVVDIKEVINWGLSGPMLRASGIQWDLRKVDNY
Arabidopsis         DYFLTEVVEYQKLITRNPIFLERVEGVGIIGGEEAINWGLSGPMLRASGIPWDLRKIDRY
Solanum             DYFLTGVAEYQKLITRNPIFLERVEGVGIIGRDEALNWGLSGPMLRASGIEWDLRKVDHY
Vitis               DYFLTGVAEYQKLITRNPIFLERVEGVGIIGVEEAINWGLSGPMLRASGIQWDLRKVDHY
                    ****  : **::***.**** .*** :* :  .*.:**********.**: *****:* *
```

```
Synechocystis      ECYDELDWEVQYETAGDCFARYLVRIREMRESVKIIRQALKAMPGGPYENLEAKRLQEGK
Physcomitrella     ECYDELDWQIQWQKEGDSLARYLVRIGEMKESIKIIQQALKSIPGGPYENLEARRLQRGK
Sorghum            ESYNQFDWKVQWQKEGDSLARYLVRVGEMRESIKIIQQAVEKIPGGPYENLEARRFKKAK
Brachypodium       ESYNQFGWKVQWQKEGDSLARYLVRIGEMRESIKIIQQAVEKIPGGPYENLEVRRFKKEK
Oryza              ESYNQFDWKVQWQKEGDSLARYLVRIGEMRESIKIIQQAVEKIPGGPYENLEVRRFKKAK
Glycine            ECYEEFDWEVQWQKEGDSLARYLVRIGEMMESIKIIQQALEGIPGGPYENLEIRCFDREK
Arabidopsis        ESYDEFEWEIQWQKQGDSLARYLVRLSEMTESIKIIQQALEGLPGGPYENLESRGFDRKR
Solanum            ESYDEFDWQVQWQREGDSLARYLVRIGEMTESIKIIQQALEGIPGGPYENLEMRRFDRLK
Vitis              ECYDEFDWEVQWQKEGDSLSRYLVRIGEMVASIKIIQQALEGIPGGPYENLEIRCFDRAR
                   *.*::: *::*::  **.::*****: **  *:***:**:: :********* : :.. :

Synechocystis      KSEWNDFQYQYIAKKVAPTFKIPAGEHYVRLESGKGELGIFIQGNDDVFPWRWKIRSADF
Physcomitrella     KSEWNNFEYQFISKKPSPTFKLPKQEHYIRVEAPKGELGVFLIGDDSVFPWRWKIRPPGF
Sorghum            NPEWNDFEYRFLGKKPSPNFELSKQELYVRVEAPKGELGIYLVGDDSLFPWRWKIRPPGF
Brachypodium       NSEWNDFEYRFLGKKPSPNFELSKQELYVRIEAPKGELGIYLVGDDGLFPWRWKIRPPGF
Oryza              NSEWNDFEYRFLGKKPSPNFELSKQELYARVEAPKGELGIYLVGDDSLFPWRWKIRPPGF
Glycine            EPEWNEFEYRFISKKPSPTFELPKQELYVRIEAPKGELGIFLIGDQNGFPWRWKIRPPGF
Arabidopsis        NPEWNDFEYRFISKKPSPTFELSKQELYVRVEAPKGELGIFLIGDQSGFPWRWKIRPPGF
Solanum            DPEWNDFEYRFISKKPSPTFELSKQELYVRVEAPKGELGIFLIGDQSVFPWRWKIRPPGF
Vitis              DPELNDFEYRFISKKPSPTFELSKQELYVRVEAPKGELGIFLIGDQNVFPWRWKIRPPGF
                   . * *:*:*:::.** :*.*::   * * *:*: *****::: *::. ********  .*

Synechocystis      NNLQILPHILKGVKVADIMAILGSIDIIMGSVDR
Physcomitrella     INLQILPQLVKGMKLADIMTILGSIDIIMGEVDR
Sorghum            INLQILPQLVKKMKLADIMTILGSIDIIMGEVDR
Brachypodium       INLQILPQLVKKMKLADIMTILGSIDIIMGEVDR
Oryza              INLQILPQLVKKMKLADIMTILGSIDIIMGEVDR
Glycine            INLQILPQLVKRMKLADIMTILGSIDIIMGEVDR
Arabidopsis        INLQILPELVKRMKLADIMTILGSIDIIMGEVDR
Solanum            INLQILPQLVKRMKLADIMTILGSIDIIMGEVDR
Vitis              INLQILPQLVKRMKLADIMTILGSIDIIMGEVDR
                    ******.::* :*:****:**********.***
```

Supplemental Table S5. Multiple alignment of Ndh48 (PNSB1) amino acid sequences in embryophytes. Sequences from *Physcomitrella patens* (PHYPADRAFT_106212), *Oryza sativa* (Os08g0276100), *Brachypodium distachion* (BRADI_3g19630*), Populus trichocarpa* (POPTR_0001s03600g), *Arabidopsis thaliana* (AT1G15980) and *Glycine max* (GLYMA_08G218800) were obtained and aligned as described in the legend for Supplemental table 2.

```
Physcomitrella      MSAMAAQVVL-----GAGLANCPQCSICVTRATSSLSHSNPSKLQLRAFGTRTNVRLENA
Oryza               MQTPTMSTSMATPAKLP---SPPS--LPIARQ---------CCCHL----LQLGRRG--G
Brachypodium        MQTPTMYTSMAARATSA--AANLP--APPPRQ---------CYYHL----LPPAGRR--S
Populus             ----MASTSLLPKTISPFLTNPPP--LPSTHFTSKPSFFNPSADHH----LPCTRKP---
Arabidopsis         ---MASSLPLLPKPISPFFKTPPF--STSKPLV-----FL---NFQ----TRLTSRSSDV
Glycine             MAATYYLLPTSPKTFSPFLRNPPS--IPSGHHVSLLGSLL---DYP----FHCSSRRGNN
                                                                           :

Physcomitrella      SS--VVVSAKKGWFDDPFDYGADDEEDTMGELMSQGPQGAEDPRPARDPDSESGYLDFPA
Oryza               AGVARASAKKKNPWLDPFDDGPDDEFDY-RGAFSGG-KQEEDPRPPEDPANPYGFLRFPA
Brachypodium        VWALSASPKKKNPWLDPFDDGPDEDFDY-TGVYSGG-KQDEDPRPPEDPSNPYGFLRFPM
Populus             -SILTPYAKKKNPWIDIFDDGEDLDMEY-GSLFVDG-KQDEDTRPVDNPNNPYGFLKFPK
Arabidopsis         S---VNLKKKNNPWLDPFDSGEDPDNEY-GSLFADG-KQDEDPRPPDNPDNPYGFLKFPK
Glycine             SVEANCNAKKKNPWLDPFDDGEDPEMEY-GSLFADG-KQEEDPRPPDDPDNPYGFLKFPS
                             *:. : * ** * * : :        * :  ** **  :* .  *:* **

Physcomitrella      GFMPEVASLGILIRNDVRRCLCMISGGVYENLLFFPVIQLLKNRYPGVRIDVMATPRGKQ
Oryza               GYNPELDSLASKVRRDVRRACCVVSGGVYENVLFFPVVQLLRDRYPGVVVDVVASARGKQ
Brachypodium        GYNPELDSLASKVRGDVRRACCVVSGGVYENVLFFPVVQLLKDRYPGVLIDVVASARGKQ
Populus             GYNVEVAQLGLKIRGDVRRCCCMISGGVYENLLFFPVIQMLKDRYPGILVDVLASDRGKQ
Arabidopsis         GYTVELASLPLKIRGDVRRCCCVISGGVYENLLFFPTIQLIKDRYPGVQVDILTTERGKQ
Glycine             GYSVEIASLALKVRGDVRRCCCVISGGVYENLLFFPAIQLIKDRYPGVQIDVVASERGKQ
                    *:  *: .*   :* ****. *::*******:****.:*::::****: :*:::: ****

Physcomitrella      AYEMNKNVRKAWVHPVDDQFLRPVDFTETVGKIKGEYYDLLVSTKLAGLGQSIFFWLASV
Oryza               VYEMCKNVRYADVYDPDDDWPEPAEYTHQLGVLKNRYYDLIISTKLAGIGHALFLFMSSA
Brachypodium        VYEMCKNVRYANVYDPDDDWPEPAEYTHQLGVLKNRYYDMILSTKLAGTGHALFLFMSSA
Populus             CYELNKNVRWANVYDPDG-DPEPAIYTDMIGVLKNRYYDLVLSTKLAGLGHASFMFMSSA
Arabidopsis         TYELNKNVRWANVYDPDDHWPEPAEYTDMIGLLKGRYYDMVLSTKLAGLGHAAFLFMTTA
Glycine             TYELNKNVRWANAYDPDDEFPEPAEYTDMVGVLKNRYYDMVLSTKLAGLGHAAFLFMTTA
                     **: **** * .:  *.   .*. :*. :* :*..***:::****** *:: *:::::.

Physcomitrella      RNKVSYTYPDVNAAGAAKFLDIAIKAPQLELAESGFNMYAEMIEELSQMGKNVPKTEVPP
Oryza               RDKVGYVYPNVNSAGAGLFLTEMFRPPTTNLADSGYNMYQDMLEWIGRPAKGVPEHPVPP
Brachypodium        REKVGYVYPNVNGAGAGLFLTEMFKPPTTNLSDGGYNMYQEMLEWLGRPGKGVPQQPIPP
Populus             RDKVSYIYPNVNAAGAGLLLTETFTPDSANLSEGGYHMYHQMLDWLGRPIYNVPRQPVPP
Arabidopsis         RDRVSYIYPNVNSAGAGLMLSETFTAENTNLSELGYSMYTQMEDWLGRPFRSVPRTPLLP
Glycine             RDRVSYIYPNVNAAGAGLLLSETFVPDSQNLSDGGYNMYHQMVDWLGKPFREVPRQPVPP
                    *::*.* **:**.***. :*   :     :*:: *: ** :* : :.:    **.  : *

Physcomitrella      LEVGIGSKVKAYVEAKYREAGVREGEFLVFHGIECDSSASMTSKGDKDCLLPLSMWAEIA
Oryza               LRVSISKKLRAFVEDKYSRAGVEKGKFVVVHGIASDSFANMRSRGDDDCLLPLEHWAEIA
Brachypodium        LRVSISKKLRGIVEDKYSRAGVEKGKFVVVHGIASDSVANMTSRGDDDCLLPLEQWAEIA
Populus             LRVSLSRKLKQYVEAKYRAAGAEKGKYIVIHGIESDSKASMQSRGDTDSLLPLEVWDQIA
Arabidopsis         LRVSISRKVKEVVAAKYRNAGAVTGKFIVIHGIESDSKASMQSKGDADSLLSLEKWAKII
Glycine             LRVSISKKLKEVVEAKYEKAGAKKGKYVVIHGIKSDSKASMQSRGDPDSLLPIEVWAEIA
                    *.*.:. *::  *  **  **.  *:::*.*** .** *.* *:** *.** :. * :*
```

```
Physcomitrella    KSTS-----DKVVFVIPNEKWRRKVKEI-CGENAHIVFITTPGQLGALINASKGVVTTNT
Oryza             KEISSEDNGLKPLFVIPHQKHREEVEET-VGKDTNILFITTPGQLTCLINDSVGVVATNT
Brachypodium      KAISSQGNGLRPLFVMPHQKHREEIEDI-VGRETSYLFITTPGQLTCLINDSAGVVATNT
Populus           DAIS----GFKPVFVIPHEKERENVEEIIYNEDIGILFITTPGQ-ATLINDSAGVIATNT
Arabidopsis       KGVR----GFKPVFVIPHEKERENVEDF-VGDDTSIVFITTPGQLAALINDSAGVIATNT
Glycine           DVIR----DVTPLFVIPHEKERENVEEI-FSEDASIVFITTPGQLAALINDSAGVIATNT
                  .           :**:*::* *.::::   . :   :*******   *** * **::***

Physcomitrella    AALQIAIALKKSTVALFASQEKANLFIPDYAK-DACAMVASKTGKLCGLDLKAATMAVST
Oryza             AAVQLANARDKPCVALFSSKEKARLFLPYLEEKKGCTVVASETGKLIDIDVEAVKKAVKE
Brachypodium      AAVQLANARDRPCVALFSSKEKAKLFLPYAEDKKSCTVVASATGKLADIDIEAVKNAVKE
Populus           AAIQLANAREKPSIALFGSEEKGKVFVPNAEE-KKCIIVSSKTGKLKDIDVGAVKQAMQI
Arabidopsis       AAIQLANARDKPCIGLFSSEEKGKLFVPYAEEKSNCVIIASKTGKLADIDIGTVKNAMQV
Glycine           TAVQLANAREKPCIALFCSEEKGNKFVPRAEE-KKCIIISSKTGKLIDIDVEAVKNAVQT
                  :*:*:* * .:  :.** *:**.. *:*   . . * :::* **** .:*: :.. *:.

Physcomitrella    IAKEALVAA---
Oryza             FKAAPSVALAQT
Brachypodium      FEPAPSFALAQK
Populus           FDMSLALV----
Arabidopsis       FEGSLALV----
Glycine           FNLSPALV----
                  :     ..
```

Supplemental Table S6. Forward and reverse primers used for cloning of bimolecular fluorescence complementation (BiFC) constructs.

Restriction enzyme sites are underlined and translational start codon is marked by bold italic. Frw = forward primer, rev = reverse primer. In order to get proper fusion to C-terminal YFP half, reverse primer lacks a stop codon. RE = restriction enzyme. The cDNA clones were obtained from the Arabidopsis Biological Resource Center (ABRC). All primers were purchased from Sigma Aldrich.

**Construct(s)**	**AGI-code**		**Primer sequences**	**RE-sites**
pSPYCE.NDH-S	AT4G23890.1	Frw<br>Rev	5´- CTGCAGAGGATCC***ATG***GCGACTTCTTCGATCAC -3´<br>5´- CTGCAGACCCGGGTGGTGCTGCCTCTTCCTTTT -3´	BamHI<br>XmaI
pSPYNE.NDH-S	AT4G23890.1	Frw<br>Rev	5´- CTGCAGAGGATCC***ATG***GCGACTTCTTCGATCAC -3´<br>5´- CTGCAGACCCGGGTGGTGCTGCCTCTTCCTTTT -3´	BamHI<br>XmaI
pSPYCE.PGR5	At2g05620.1	Frw<br>Rev	5´- CTGCAGAGGATCC***ATG***GCTGCTGCTTCGATTTC -3´<br>5´- CTGCAGACCCGGGAGCAAGGAAACCAAGCCTCT -3´	BamHI<br>XmaI
pSPYNE.PGR5	At2g05620.1	Frw<br>Rev	5´- CTGCAGAGGATCC***ATG***GCTGCTGCTTCGATTTC -3´<br>5´- CTGCAGACCCGGGAGCAAGGAAACCAAGCCTCT -3´	BamHI<br>XmaI
pSPYCE.PGRL1A	At4g22890	Frw<br>Rev	5´- CTGCAGAGGATCC***ATG***GGTAGCAAGATGTTGTTTA -3´<br>5´- CTGCAGACCCGGGAGCTTGGCTTCCTTCTGGC -3´	BamHI<br>XmaI
pSPYNE.PGRL1A	At4g22890	Frw<br>Rev	5´- CTGCAGAGGATCC***ATG***GGTAGCAAGATGTTGTTTA -3´<br>5´- CTGCAGACCCGGGAGCTTGGCTTCCTTCTGGC -3´	BamHI<br>XmaI

SUPPLEMENTAL REFERENCES

[1]Perez-Ruiz, J.M. and Cejudo, F.J. (2009). A proposed reaction mechanism for rice NADPH thioredoxin reductase C, an enzyme with protein disulfide reductase activity. FEBS Lett. 583, 1399-1402.

[2]Richter, A.S., Peter, E., Rothbart, M., Schlicke, H., Toivola, J., Rintamäki, E., and Grimm, B. (2013). Posttranslational Influence of NADPH-Dependent Thioredoxin Reductase C on Enzymes in Tetrapyrrole Synthesis. Plant Physiol. 162, 63-73.

[3]Hall, M., Mata-Cabana, A., Akerlund, H.E., Florencio, F.J., Schroder, W.P., Lindahl, M., and Kieselbach, T. (2010). Thioredoxin targets of the plant chloroplast lumen and their implications for plastid function. Proteomics 10, 987-1001.

[4]Valerio, C., Costa, A., Marri, L., Issakidis-Bourguet, E., Pupillo, P., Trost, P., and Sparla, F. (2011). Thioredoxin-regulated beta-amylase (BAM1) triggers diurnal starch degradation in guard cells, and in mesophyll cells under osmotic stress. J. Exp. Bot. 62, 545-555.

[5]Seung, D., Thalmann, M., Sparla, F., Abou Hachem, M., Lee, S.K., Issakidis-Bourguet, E., Svensson, B., Zeeman, S.C., and Santelia, D. (2013). Arabidopsis thaliana AMY3 Is a Unique Redox- regulated Chloroplastic alpha-Amylase. J. Biol. Chem. 288, 33620-33633.

[6]Balmer, Y., Koller, A., del Val, G., Manieri, W., Schurmann, P., and Buchanan, B. (2003). Proteomics gives insight into the regulatory function of chloroplast thioredoxins. Proc. Natl. Acad. Sci. U. S. A. 100, 370-375.

[7]Mikkelsen, R., Mutenda, K., Mant, A., Schurmann, P., and Blennow, A. (2005). alpha-Glucan, water dikinase (GWD): A plastidic enzyme with redox-regulated and coordinated catalytic activity and binding affinity. Proc. Natl. Acad. Sci. U. S. A. 102, 1785-1790.

[8]Yamaryo, Y., Motohashi, K., Takamiya, K., Hisabori, T., and Ohta, H. (2006). In vitro reconstitution of monogalactosyldiacylglycerol (MGDG) synthase regulation by thioredoxin. FEBS Lett. 580, 4086-4090.

[9]Marchand, C., Le Marechal, P., Meyer, Y., and Decottignies, P. (2006). Comparative proteomic approaches for the isolation of proteins interacting with thioredoxin. Proteomics 6, 6528-6537.

[10]Skryhan, K., Cuesta-Seijo, J.A., Nielsen, M.M., Marri, L., Mellor, S.B., Glaring, M.A., Jensen, P.E., Palcic, M.M., and Blennow, A. (2015). The Role of Cysteine Residues in Redox Regulation and Protein Stability of Arabidopsis thaliana Starch Synthase 1. Plos One 10, e0136997.

[11]Braun, P., Carvunis, A., Charloteaux, B., Dreze, M., Ecker, J.R., Hill, … and Arabidopsis Interactome Mapping Co (2011). Evidence for Network Evolution in an Arabidopsis Interactome Map. Science 333, 601-607.
