# Supplementary material for: Regulation of cyclic electron flow by chloroplast NADPH‐dependent thioredoxin system
Source: Plant Direct. 2018 Nov 7;2(11):e00093. doi: 10.1002/pld3.93 (PMC6508795; doi:10.1002/pld3.93)
Supplement: Supplementary file 1 [file PLD3-2-e00093-s001.pdf]

Response letter / Manuscript Plant Direct: 2018-00183

Thank you for your valuable comments to our manuscript.

We have now revised our manuscript according to the reviewer's comments. The major corrections are listed below and the revised manuscript, in which the corrections are marked by yellow paint, is included in the submission.

Response to the reviewer's comments and revision made in the revised manuscript.

General questions:

• There is no PIFR in ndho and a diminished PFIR in pgr5 (Fig. 2b). How can this be explained? The authors fully attribute the PIFR to NDH activity. Do the authors suggest diminished NDH activity in pgr5 is a pleiotropic effect of the PGR5 mutation?
Response: It has been shown that the capacity to oxidize PSI is impaired in pgr5 mutant (e.g. Tiwari et al., 2016; Takagi and Miyake 2018) that decreases the availability of reduced ferredoxin, the substrate of the NDH complex. This is now explained in the revised manuscript on page 7.

• How exactly do the authors imagine a limitation of electron flow at Cyt b6f in ntrc? If this was photosynthetic control for example, one would expect an exaggerated ΔpH in comparison to WT, which was not observed at least in growth and high light.
Response: Limitation of electron flow between PQ pool and PSI in ntrc might be related to the same PGR5-dependent mechanism that prevents OE-NTRC pgr5 from inducing photosynthetic control in high light despite recovery of WT-level pmf. If the relevant function of PGR5 is dependent on chloroplast thiol redox state, the 40 % decrease in PGR5 content in ntrc may occur to inhibit an over-active (due to altered stromal redox state) mechanism of photosynthetic control. However, this discussion is so far mostly speculative, falls outside the scope of the current study, and it would require further experiments and lengthy discussion to adequately verify and explain. Therefore we would here prefer to merely point out this observation. "Possibly at Cyt b6f" has been removed from the sentence in the manuscript on page 11.

• How do the authors explain the extremely elevated pmf in ntrc compared to WT in low light conditions (Fig. 5e)? (Impaired CF1γ reduction?)
Response: This question was discussed in the original manuscript on page 12: "In ntrc, high steady state pmf under low light intensity (Fig. 7) was likely caused by impaired activation of the chloroplast ATP synthase (= impaired CF1γ reduction) and the Calvin-Benson cycle as previously reported (Nikkanen et al. 2016, Carrillo et al. 2016). Furthermore, exceptionally high NPQ was recorded in the ntrc line, especially at low light (Suppl. Fig. S5)." This chapter is on page 13 in revised manuscript.

• It is still not clear to me how NDH is supposed to significantly contribute to CEF during the induction phase of photosynthesis. Being highly substoichiometric with regard to PSI, it would have to sustain very high rates, which cannot be mediated by a diffusion limited PSI-NDH complex and which also contradicts the very low NDH rates measured in vivo (Trouillard et al. 2012).
Response: Of course this is a tricky question to which no one seems to have final concluding answer. As far as there is no direct assay available to measure the activity chloroplast NDH complex, it is not possible to unambiguously demonstrate its activity in vivo (high or low rate). Also In the paper by Trouillard et al. 2012 the activity of NDH was estimated indirectly: They wrote "From the kinetic analysis of the profiles shown in Fig. 2 (presenting fluorescence induction kinetics measured in tomato leaves), it is possible to derive quantitative information of the influx and outflux of electron undergone by the PQ pool, i.e. on the activity of the NDH and PTOX enzymes." It is, however, difficult

to validate, how well this influx and outflux of electrons represent specifically and only the activities of NDH and PTOX enzymes in vivo?
However, it was recently demonstrated by electron microscope analysis of single particle projections of supercomplexes that a single NDH complex can bind up to six PSI complexes (Yadav et al., 2017). If this is the case, it will solve, at least to some extent, the stoichiometric differences in the amount of NDH and PSI complexes. It might be that NDH complex is mostly needed at dark-to-light transitions as well as during fast fluctuations in light intensity, when other PSI acceptors are less active, like suggested in the current paper as well as in another recent paper by Shimakawa & Miyake (2018). This is now discussed in more detail on page 19 of the revised manuscript.

Other comments:
Introduction:
• The sentences
"...both the PGR-dependent pathway (Hertle et al., 2013; Strand et al., 2016a) and the NDH pathway (Courteille et al., 2013) have been proposed to be subject to thiol-regulation by chloroplast thioredoxins." (page 4)
and
"Moreover, redoxregulation of both CEF pathways has been previously reported (Courteille et al., 2013; Hertle et al., 2013; Strand et al., 2016a)." (page 5)
are redundant.
Response: This is corrected in revised manuscript on pages 4–5: the latter sentence has been deleted.

Results:
• Please consistently add letters when referring to subfigures, this would facilitate readability a lot (e.g. Fig. 1a instead of just Fig. 1).
Response: Subfigure letters have been added throughout the revised manuscript.

• The order of figures and subfigures is sometimes not matching their first mention in the text.
Response: The order of figures is matched with the text. In some cases the subfigures are not always mentioned in the same order in the text that they are presented in the figures. This is the case with the figures containing large datasets and subfigures (Fig. 5, 6, 7, 8) from the experiments made with lines having both ndho and pgr5 mutant background. These mutants have different WT background and for clarity and for easy comparison the results are presented in different subfigures side by side (e.g. pmf (A and B), gH+ (C and D), etc.). Therefore sometimes e.g. Fig. 5C is mentioned before Fig. 5B.

• It should be mentioned and discussed that LHCII phosphorylation in HL is more pronounced in ntrc compared to WT (Fig. 4h).
Response: This is now mentioned on page 9 of the revised manuscript.

• Suppl. Table S1 could be described and discussed in more detail.
Response: Table S1 is now described on page 7 of the revised manuscript.

• There is a wrong reference to Fig. 4 in the paragraph describing the pmf kinetics, it should probably be Fig. 5 (page 9).
Response: This is now corrected on page 10 of the revised manuscript.

• There is a wrong reference to Fig. 5 and 6 regarding the P700 oxidation, it should probably be Fig. 6 and 8 (page 10)
Response: This is now corrected on page 10 of the revised manuscript.

• "P700 oxidation was significantly enhanced" (page 10): P700 oxidation is faster in OE-NTRC compared to WT, but I doubt that Y(ND) after 60s significantly differs from WT (in particular Col-gl1).
Response: This is now corrected on page 10 of the revised manuscript.

• "At steady state, pmf in OE-NTRC pgr5 plants did not differ from pgr5 plants, while proton conductivity of the thylakoid membrane was drastically higher even in comparison to pgr5." (page 11): Does this refer to the proton conductivity before or after 60s ("steady state")? The difference after 60s does not seem so drastic to me (Fig. 5d). The difference before 60s seems to be analogous to the gH+ kinetics in OE-NTRC ndho (Fig. 5c).
Response: These sentences are now reformulated on page 11 of the revised manuscript.

• "At steady state the pmf was comparable to WT, but contribution of ΔpH to total pmf was slightly diminished". (page 11): This is only true for growth light and high light, not for low light! (Fig. 5e)
Response: The text on page 11 dealt with the pmf of ntrc under growth light illumination. The pmf under low light (Fig. 5E and Fig. 7A) is dealt on page 13: "In ntrc, high steady state pmf under low light intensity (Fig. 5E, Fig. 7A) was likely caused by impaired activation of the chloroplast ATP synthase and the Calvin-Benson cycle as previously reported (Nikkanen et al. 2016, Carrillo et al. 2016)". The sentence on page 11 has been modified in order make clear that it refers to growth light conditions.

• "Notably, the overexpression of NTRC in ndho background reverted these changes observed in OE-NTRC plants to levels comparable to ndho knockout plants, except for the increased PSII yield during dark-to-low light transitions." (page 11/12): Although the kinetics observed in OE-NTRC ndho (except for Y(II)) are similar to ndho, the levels are not, they seem mostly even lower/higher than in ndho compared to OE-NTRC (Fig. 7a and b/Fig. 8).
Response: This sentence is reformulated in revised manuscript on page 12.

• There is a wrong reference to Fig. 8 regarding gH+, it should probably only be Fig. 7 (page 12).
Response: This is corrected in revised manuscript on page 12.

Discussion
• There is a wrong reference to Fig. 3 regarding increased dark-reduction of PQ in short day-grown ntrc, it should probably be Fig. 4 (page 15).
Response: This is corrected (Fig. 4G) in revised manuscript on page 15.

• There is a wrong reference to Fig. 3 regarding the effect of NTRC on NDH-dependent CEF, it should probably be Fig. 2, Fig.4, Fig. 5, Fig. 6, Fig. 7, Fig. 8 (page 16).
Response: The references to figures are corrected in revised manuscript on page 17.

• Avenson et al. 2005 (page 17 and 19) do not suggest a direct association of PGR5 with the ATPase in their discussion.
Response: Citations to Avenson et al. 2005 and Armbruster et al. 2017 are omitted on pages 17 and 20 of the revised manuscript.

Figures
• Figure 1:
Maybe include a label which line the samples originate from (A: WT/B: OE-NTRC/C: WT)
Response: Figure A and B have been combined (now Fig. 1A in revised manuscript) and the plant line marked under the immunoblots.

• Figure 2:
a and b: Maybe add a time scale to the magnifications.
It is not clear why in Fig. 2a and b the authors chose to show the whole experiment while only showing the PFIR phase in Fig. 2c.
Response: Time scales are included into the magnifications. Whole fluorescence measurement is included in Fig. 2C and the measurement described on page 23.

• Figure 4a to f:
Maybe add a legend and format consistently regarding the black-red overlay.
Response: It is now indicated in Fig. 4A that black and red curves represent dark-adapted and FR-pre-illuminated leaves, respectively.

• Figure 5e: The figure legend should include a description of the statistics.
Response: Statistics is now included in the legend of Fig. 5E.

• Figure 7c: Black symbols should probably be triangles, not circles.
Response: This is now corrected in Fig. 7C.

Sincerely,

Eevi Rintamäki
